# Peptide‐Enabled Nanoplatforms for Malaria and Leishmaniasis: From Intracellular Targeting to Translational Diagnostic Perspectives

**DOI:** 10.1002/cmdc.70493

**Published:** 2026-09-16

**Authors:** Christian S. Carnero Canales, Ana Clara Lunardi Yagi, Túlio Custódio Reis, Jessica Ingrid Cazorla, Jade Rojas Villar, Marcia A. Graminha, Roxana Yesenia Pastrana Alta, Fernando Rogério Pavan

**Affiliations:** ^1^ BIOMET Laboratorio de Química Bioinorgánica en Medicina Medioambiente y Tecnología Facultad de Ciencias de la Universidad Nacional de Ingeniería Lima Peru; ^2^ School of Pharmaceutical Sciences São Paulo State University (UNESP) Araraquara Brazil; ^3^ General humanidades Universidad Continental (UC) Arequipa Perú; ^4^ School of Pharmacy Biochemistry and Biotechnology Universidad Católica Santa María (UCSM) Arequipa Perú

**Keywords:** antimicrobial peptides, cell‐penetrating peptides, leishmaniasis, malaria, nanocarriers, nanoplatforms

## Abstract

Malaria and leishmaniasis continue to impose a substantial health burden, while current therapeutic and diagnostic strategies remain limited by toxicity, prolonged regimens, drug resistance, and restricted field applicability. This review examines recent advances in peptide‐enabled nanoplatforms for both diseases, with emphasis on intracellular targeting, drug delivery, and diagnostic innovation. Antimicrobial peptides, cell‐penetrating peptides, peptoids, mimotopes, and peptide‐functionalized nanocarriers have been explored to improve parasite targeting, intracellular delivery, pharmacokinetics, and antiparasitic efficacy. In parallel, peptide‐ and aptamer‐based nanosensors have expanded the detection of clinically relevant biomarkers, including HRP2, pLDH, GP63, KMP‐11, and kDNA, highlighting their potential for sensitive and modular diagnostics. Despite these advances, translation remains constrained by limited in vivo validation, insufficient pharmacokinetic standardization, incomplete toxicity profiling, poor batch reproducibility, protein corona‐associated variability, and scarce validation under field‐relevant conditions. Peptide‐enabled nanoplatforms are therefore most likely to succeed when aligned with stage‐specific parasite biology, realistic host cell‐targeting requirements, and scalable implementation pathways in endemic settings.

## Graphical abstract

Peptide‐enabled nanoplatforms offer multifunctional strategies for the treatment and diagnosis of malaria and leishmaniasis. Liposomal, polymeric, and metallic nanocarriers, together with cell‐penetrating peptides and parasite‐derived mimotopes, can improve targeted drug delivery, stability, bioavailability, and intracellular localization. These peptide‐based systems can also support the recognition and detection of disease‐specific biomarkers. By integrating therapeutic activity, targeting, cargo delivery, and molecular recognition, peptide‐enabled nanoplatforms provide versatile approaches for overcoming biological barriers associated with both parasitic diseases.

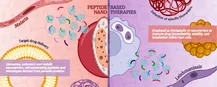

## 1 Introduction

Malaria control continues to be challenged by fluctuating transmission patterns, uneven access to interventions, and persistent reliance on a limited range of antimalarial drugs [1]. This situation has become more concerning with the emergence of partial resistance to artemisinin in East Africa, where kelch13‐related variants have been identified in *Plasmodium falciparum* populations in Uganda [2]. At the same time, the performance of histidine‐rich protein 2 (HRP2)‐based rapid tests is compromised by persistent antigenemia after treatment and by pfhrp2/3 deletions, which has prompted a progressive move toward enzymatic markers such as *Plasmodium* lactate dehydrogenase (pLDH) and *Plasmodium* glutamate dehydrogenase (pGDH) as alternative targets [3, 4, 5]. In leishmaniasis, the coexistence of cutaneous, mucosal, and visceral forms continues to complicate the diagnosis and treatment in endemic regions [6]. Although liposomal amphotericin B remains one of the most effective therapeutic options, its use is constrained by cost, toxicity, and the need for intravenous administration, which limits accessibility [7]. These limitations are further reinforced by parasite biology, as *Leishmania* survives within macrophages by altering complement activation, phagolysosomal maturation, and immune signaling pathways [8]. As a result, nanomedicine has been explored to improve drug delivery to infected cells while reducing systemic exposure [9].

Quantifying this joint burden clarifies why both diseases justify a shared research effort. In the Global Burden of Disease 2021 analysis, neglected tropical diseases and malaria together produced 71.63 million disability‐adjusted life years (DALYs) and a global incidence of 4,259.54 cases per 100,000 population, with the highest rates concentrated in low sociodemographic index regions and in children under 5 years of age [10]. Within the group of vector‐borne parasitic diseases of poverty, malaria shows the highest age‐standardized prevalence (2,336.8 per 100,000) and the highest age‐standardized DALY rate (806.0 per 100,000) [11]. Control efforts have produced measurable gains—the WHO African Region recorded a 40% decrease in malaria incidence and a 60% reduction in mortality between 2000 and 2022 [12]—yet the absolute case load remains near historic highs and progress has plateaued in the highest transmission settings [13]. Leishmaniasis follows a parallel trajectory: Age‐standardized incidence, mortality, and DALY rates for visceral leishmaniasis declined between 1990 and 2021, but mortality remains highest in children under five and the disease persists as an under‐recognized problem across Latin America, East Africa, the Middle East, and South Asia [14]. Cutaneous forms contribute a burden dominated by years lived with disability rather than mortality, reflecting disfigurement and stigma rather than lethality [15], and an estimated 0.7–1 million new cases arise annually across roughly 100 endemic countries [16].

Reviewing malaria and leishmaniasis together is not a matter of convenience but of shared biology and shared translational constraints. Both are caused by protozoa that spend their pathogenic phase inside host cells—hepatocytes and erythrocytes for *Plasmodium*, mononuclear phagocytes for *Leishmania*—so that any therapeutic agent must cross at least two membrane systems before reaching its target, a requirement that conventional small‐molecule pharmacology addresses poorly [17]. Genome‐wide screening in host cells has shown that the intracellular development of *P. falciparum* and related hemoparasites converges on overlapping host metabolic dependencies; this finding is relevant to host‐directed delivery in malaria but should not be extrapolated directly to Leishmania [18]. The two diseases are also co‐endemic across large areas of sub‐Saharan Africa, South Asia, and South America, where both are recurrent causes of prolonged undifferentiated fever and are routinely considered in the same differential diagnosis [19]; experimental co‐infection confirms reciprocal immune modulation, with acute *P. berghei* infection reducing lesion size and parasite burden in *Leishmania major*‐infected mice while altering monocyte recruitment [20]. Climate‐driven expansion of the ranges of *Anopheles* and phlebotomine vectors is projected to widen this geographical overlap [21].

The therapeutic and diagnostic bottlenecks are likewise common to both diseases, and they are the reason why a joint analysis is informative. Both rely on a small number of drug classes whose useful life is being shortened by resistance. Artemisinin partial resistance is now established in multiple East African countries, while antimonial and miltefosine failure is widespread in leishmaniasis [22, 23]. Both face important limitations in point‐of‐care diagnosis, although the underlying limitations differ. pfhrp2/3 deletions compromise HRP2‐based malaria tests [24], while existing cutaneous leishmaniasis (CL) assays still fail to meet the WHO target product profile for sensitivity, species typing, and cost [25]. Both are diseases of poverty in which affordability governs adoption: First‐line treatment of a single visceral leishmaniasis patient in Ethiopia costs approximately US$104.7, rising to US$331.9 for second‐line therapy, an expenditure sustained largely by external donors rather than national budgets [26]. They are also targets of elimination programs whose gains are fragile and depend on tools that are simpler, cheaper, and more stable than those currently available while remaining chronically underserved by commercial drug discovery [27, 28]. These converging constraints define the design space that peptide‐enabled nanoplatforms are being developed to occupy.

In parallel, peptides have gained attention due to their capacity for sequence‐specific design, enabling membrane interaction, receptor recognition, and cargo transport. However, their application remains constrained by in vivo instability and potential off‐target effects when not adequately protected [29]. To address these limitations, recent approaches increasingly combine peptide design with engineered delivery systems capable of overcoming intracellular barriers, modulating release profiles, and improving subcellular targeting [30]. Evidence also indicates that the performance of peptide‐functionalized nanoparticles depends on peptide properties, nanoparticle composition, ligand density, and the selectivity of cell interactions [31]. These strategies are already being explored in malaria, where phosphatidylserine‐targeted erythrocyte membrane nanodecoys carrying artemether have shown preferential interaction with infected erythrocytes and improved survival in experimental models [32]. Comparable developments have been reported in diagnostics, including peptide‐based fluorescent systems for *P. falciparum* and peptide–carbon nanomaterial sensors for leishmaniasis, both showing improved analytical performance [33, 34]. This review examines the integration of peptide‐based strategies with nanoplatforms for malaria and leishmaniasis, focusing on their role in intracellular targeting, drug delivery, and diagnostic innovation. Particular emphasis is placed on how peptide properties and nanomaterial design jointly influence selectivity, pharmacokinetics, and translational potential. By critically analyzing recent advances, we aim to identify key limitations, design principles, and emerging opportunities for the development of clinically relevant and field‐deployable technologies in endemic settings.

## 2 Malaria (*Plasmodium* spp.)

Malaria is a vector‐borne parasitic disease caused by protozoa of the genus *Plasmodium* and transmitted to humans through the bite of infected female *Anopheles* mosquitoes [35]. Among the species infecting humans, *P. falciparum* and *P. vivax* account for most infections worldwide, with *P. falciparum* responsible for most severe cases and malaria‐related mortality [36], a burden concentrated in tropical and subtropical regions [37]. Of the many biological details of the parasite, two are decisive for the present discussion. Each developmental stage exposes a distinct set of surface ligands and intracellular compartments [38], and the spread of *kelch13*‐mediated artemisinin partial resistance has narrowed the therapeutic margin of first‐line regimens [39]. Together, they define where peptide‐guided delivery can add selectivity that small molecules alone no longer provide.

**FIGURE 1:**
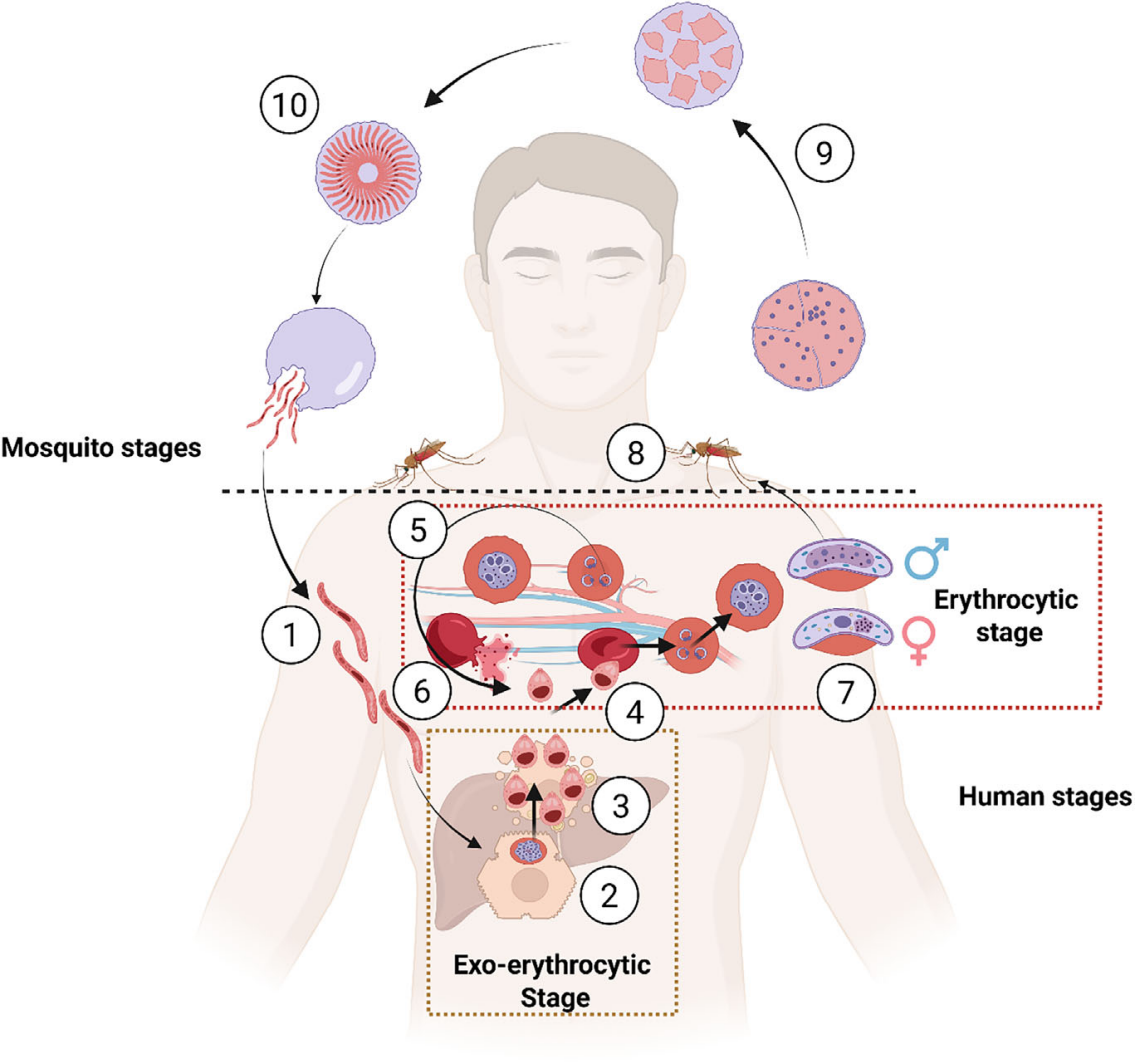
The general biological cycle of the genus *Plasmodium* in its vertebrate and invertebrate hosts. The Anopheles mosquito, the only known vector, transmits the parasite by inoculating sporozoites (approximately 50 sporozoites) (a)—the infective stage—during a blood meal (1). The exoerythrocytic cycle begins when sporozoites infect hepatocytes, where they mature and develop into schizonts (2). These schizonts rupture, releasing merozoites (3), which invade erythrocytes, thereby initiating the erythrocytic cycle (4). Within red blood cells, the parasites multiply asexually, progressing from the ring stage to the mature trophozoite (5) and finally to the schizont, which ruptures and releases new merozoites (6). Ring‐stage parasites develop into mature trophozoites and differentiate into male and female gametocytes (7). When a mosquito ingests these gametocytes during a blood meal, the sexual cycle begins (8). Fertilization in the mosquito midgut produces a zygote, which matures into an ookinete and subsequently into an oocyst (9, 10). Finally, the oocyst ruptures, releasing sporozoites that migrate to the salivary glands, rendering the mosquito infectious to a new host (Created by the author in BioRender.com) [37, 40].

The life cycle (Figure 1) alternates between an asexual phase in the human host—comprising a hepatic (exoerythrocytic) stage and an erythrocytic stage that produces the clinical manifestations—and a sexual, sporogonic phase in the mosquito [38, 41]. The following subsections therefore consider only those features of each stage that constitute actionable targets for peptide‐based delivery or detection.

### 2.1 Hepatic Stage

After entering the bloodstream, *Plasmodium* sporozoites migrate to the liver, where recognition is driven by electrostatic interactions between the positively charged circumsporozoite protein (CSP) and negatively charged glycosaminoglycans on hepatic stellate cells lining the fenestrated sinusoids [42]. Only a small proportion of sporozoites crosses the sinusoidal barrier to reach hepatocytes, yet each infected hepatocyte releases approximately 3 × 10^4^ merozoites [43]. This amplification step, combined with the very low sporozoite inoculum required to establish infection, makes the hepatic stage the point at which intervention is most efficient: A delivery system that reaches hepatocytes before schizogony acts on the smallest parasite population of the entire cycle.

Several peptide‐based strategies have been explored to block liver‐stage infection. Cha et al. [44] used a phage display library to identify HP1, a hepatocyte‐binding mimotope corresponding to a ~50 kDa sporozoite ligand later identified as phospholipid scramblase. HP1 interacts with a putative ~160 kDa hepatocyte receptor, carbamoyl‐phosphate synthetase 1, and immunization with the peptide conferred partial protection against *P. berghei* in mice. Antibodies raised against HP1 also inhibited *P. falciparum* invasion of human hepatocytes in vitro, supporting its potential as a ligand–receptor decoy. A previous work from the same group identified P39, a peptide selected for high‐affinity binding to Kupffer cells, which serve as a key bottleneck for liver‐stage entry. P39 interacts with CD68, a surface molecule exploited by sporozoites, and its presence reduces hepatic invasion [45]. In a related approach, Yang et al. [46] showed that the R1 peptide decreased *P. falciparum* sporozoite entry into hepatocytes. R1 also inhibits apical membrane antigen 1 (AMA1), linking liver‐stage inhibition with activity in the erythrocytic cycle.

Peptide conjugation strategies have also been applied to enhance the delivery and selectivity of antimalarial drugs. Aguiar et al. [47] conjugated primaquine (PQ) to two cell‐penetrating peptides (CPPs), transportan and the related transportan 10 (TP10), using a C4 linker. The conjugates were evaluated in Huh‐7 hepatocytes infected with luciferase‐expressing *P. berghei* sporozoites, quantifying the parasite load at 48 h postinfection. PQ‐C4‐transportan and PQ‐C4‐TP10 exhibited greater inhibitory activity than the free CPPs or PQ alone, with the most pronounced effect observed at 10 μM. These findings illustrate how peptide carriers can improve the hepatic delivery of small‐molecule antimalarials, a strategy that could be further refined using nanostructured vehicles to enhance stability, biodistribution, and intracellular trafficking.

### 2.2 Erythrocytic Stage

After leaving the liver, merozoites invade erythrocytes by sequentially interacting with surface proteins, including members of the merozoite surface protein family (MSP‐1 to MSP‐10). Among them, MSP‐1 is the most abundant and its processed fragments bind heparin‐like molecules on the erythrocyte surface, facilitating the initial attachment required for invasion [48]. Understanding these host–parasite interactions has become essential for the design of new therapies and targeting strategies, particularly those integrating delivery‐enhancing platforms [49, 50]. One promising approach is the use of peptide–drug conjugates (PDCs). Palombi et al. [51] developed PDCs linking primaquine to PDIP, a CPP derived from human platelet factor 4. The conjugates incorporated either disulfide or trioxolane linkers, enabling responsive release of PQ under reductive or heme‐rich conditions characteristic of infected erythrocytes. This design improved the intracellular accumulation of the drug while reducing exposure to noninfected cells. The most active conjugates reached IC_50_ values near 4 μM, demonstrating how human‐derived peptides can serve as selective carriers to enhance antimalarial activity during the blood stage, with implications for overcoming resistance.

Peptide‐based delivery has also been used to examine the mechanistic consequences of CPP–drug conjugation. Aguiar et al. showed stronger binding to both zwitterionic and anionic membranes compared to TP10 alone but also induced marked membrane disruption, particularly in membranes mimicking noninfected erythrocytes. This lytic behavior was attributed to the hydrophobic quinoline core of CQ, which facilitates deeper insertion into lipid bilayers and transforms the cell‐penetrating property of TP10 into a damaging interaction. These findings highlight a key consideration for peptide–drug designs: Improved potency may be counterbalanced by increased cytotoxicity, emphasizing the importance of detailed biophysical and biological assessment prior to translational application [50].

As parasitemia increases, *P. falciparum* can progress to severe malaria, the most life‐threatening clinical form. This progression is driven by the expression of adhesion proteins such as PfEMP1 on infected erythrocytes, enabling cytoadherence to the microvascular endothelium and escape from splenic clearance [36]. Cytoadherence promotes vascular congestion, inflammation, and tissue ischemia, ultimately causing multi‐organ dysfunction involving the brain, kidneys, lungs, and liver [43]. In this context, Silva et al. [52] tested modified derivatives of human angiotensin II, including VIPF and the cyclic peptide Ang II‐SS, in murine models of *P. berghei* ANKA infection, which replicate the severe erythrocytic pathophysiology of *P. falciparum*. Both peptides reduced parasitemia and increased survival, with Ang II‐SS demonstrating superior proteolytic resistance and efficacy. Importantly, the derivatives retained antiplasmodial activity while lacking the vasoconstrictive effects of native angiotensin II, illustrating how targeted structural modification can convert endogenous peptides into safe and effective antimalarial agents.

At the organelle level, Somsri et al. [53] evaluated the mitochondria‐penetrating peptide (Fxr)^3^ against erythrocytic stages of *P. falciparum*. The peptide showed no toxicity toward L929 fibroblast cells and inhibited parasite development, preventing progression to trophozoites and schizonts while inducing vacuolization and chromatin condensation. Confocal microscopy confirmed localization within parasite mitochondria, linking the antimalarial effect to mitochondrial disruption [53]. These data extend blood‐stage peptide strategies beyond membrane or cargo delivery to direct parasite organelle targeting, although the evidence remains preclinical.

Taken together, the erythrocytic‐stage studies show that peptide‐based strategies can improve intracellular drug accumulation, confer proteolytic stability, and reduce off‐target exposure. Their limitation is equally clear: Most reports stop at in vitro IC_50_ values or short‐term murine parasitemia, without the pharmacokinetic, immunogenicity, or manufacturing data required to select a clinical candidate, and comparative benchmarking against non‐peptidic nanocarriers is rarely performed [54]. Section 7 examines these gaps in detail.

## 3 Leishmaniasis

Leishmaniasis is a neglected tropical disease caused by protozoan parasites of the genus *Leishmania* and transmitted by infected female phlebotomine sand flies (*Lutzomyia* in the Americas and *Phlebotomus* in Africa, Asia, and Europe) [55, 56]. Clinical presentation ranges from cutaneous leishmaniasis, the most frequent form, to visceral leishmaniasis (VL or kala‐azar), which is fatal if untreated [57]. The therapeutic repertoire is narrow and aging: Pentavalent antimonials, such as meglumine antimoniate and sodium stibogluconate, are increasingly associated with treatment failure, while amphotericin B, its liposomal formulation, miltefosine, and paromomycin remain limited by nephrotoxicity, teratogenicity, cost, or the need for parenteral administration [58, 59]. Two features of this profile are directly relevant to peptide‐enabled platforms: The drugs that work best are those already delivered in a nanostructured form, and the principal unmet need is selectivity for infected macrophages rather than intrinsic potency [60].

**FIGURE 2:**
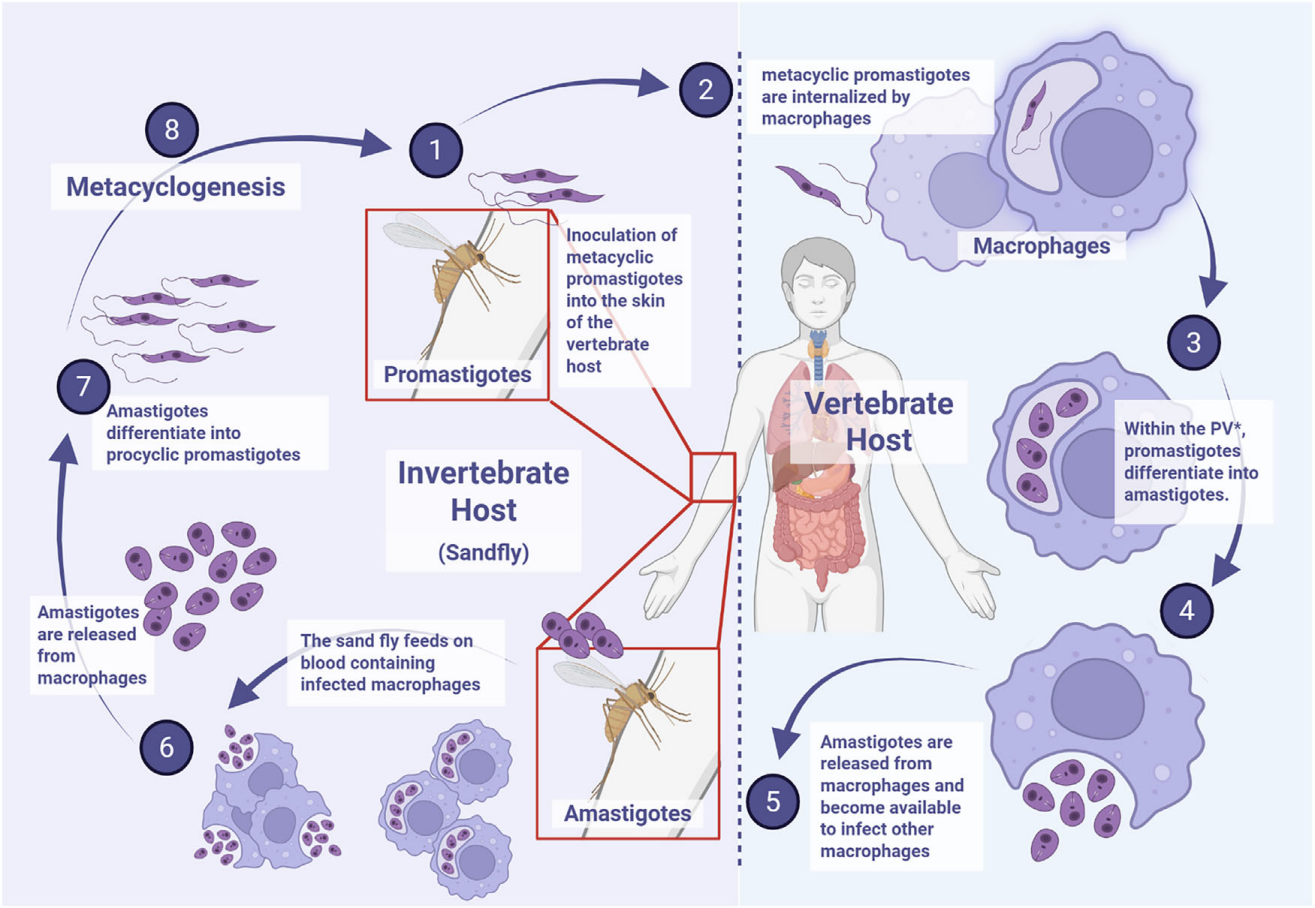
Biological cycle of *Leishmania* spp. (1) Infection begins when the sandfly vector inoculates metacyclic promastigotes into the skin of the vertebrate host. (2) These forms are phagocytosed by macrophages, (3) where they differentiate into amastigotes within the parasitophorous vacuole (PV) and replicate. (4) The macrophage eventually ruptures, releasing amastigotes that infect other macrophages across different tissues. (5) When a sandfly feeds on an infected host, it ingests parasitized macrophages, and (6) the internalized amastigotes are released into the insect midgut. (7) Inside the midgut, the amastigotes transform into procyclic promastigotes and multiply. (8) Metacyclogenesis occurs in the posterior portion of the midgut, and the resulting metacyclic promastigotes migrate to the proboscis, where they are ready to be transmitted to a new host. PV: Parasitophorous vacuole.

*Leishmania* alternates between the flagellated promastigote in the sand fly and the intracellular amastigote in the mammalian host. Metacyclic promastigotes inoculated into the skin are phagocytosed by mononuclear cells, differentiate into amastigotes, and replicate within parasitophorous vacuoles (Figure 2) [61, 62]. This intracellular lifestyle inside immune cells is simultaneously the principal obstacle to drug delivery and the reason peptide‐based carriers are attractive, since receptor‐mediated uptake and endosomal escape can be encoded directly in the peptide sequence [63, 64].

### 3.1 Host–Parasite Interactions and Clinical Manifestations

Macrophages constitute the primary host cells for *Leishmania* and represent the central niche in which the parasite establishes a long‐term infection. After inoculation into the skin, metacyclic promastigotes are initially engulfed by neutrophils and subsequently transferred to macrophages through efferocytosis within a microenvironment enriched in immunomodulatory cytokines such as transforming growth factor beta (TGF‐β), which suppresses reactive oxygen species (ROS) production and favors parasite survival [65].

Once internalized, *Leishmania* actively modulates host defenses. The metalloprotease GP63 interferes with complement activation and promotes complement receptor 3 (CR3)‐mediated uptake, a route associated with reduced inflammatory signaling; the parasite thereby inhibits phagolysosomal maturation and shifts the cytokine profile toward IL‐4, IL‐10, and TGF‐β [66, 67]. The practical consequence for drug design is that macrophages become permissive reservoirs protected by two successive membranes, so that conventional drugs reach the parasitophorous vacuole at subtherapeutic concentrations [68]. Strategies capable of delivering cargo across both barriers, rather than merely increasing systemic exposure, are therefore the central requirement.

Clinically, tegumentary leishmaniasis spans localized cutaneous lesions to destructive mucosal disease, with the phenotype largely determined by the host cell‐mediated response: Localized CL is associated with a comparatively balanced response, diffuse CL with severely impaired cell‐mediated immunity and high parasite loads [69, 70], and mucocutaneous disease with an exaggerated Th1‐mediated inflammatory response causing progressive mucosal destruction [71, 72, 73]. Because parasite burden and immunopathology vary in opposite directions across this spectrum, a single therapeutic strategy is unlikely to serve all forms—an argument for modular platforms in which the targeting peptide, the cargo, and the release trigger can be varied independently.

#### 3.1.1 Visceral Leishmaniasis (Liver and Spleen)

Visceral leishmaniasis is caused mainly by *Leishmania donovani* and *Leishmania infantum* [74], although species usually associated with cutaneous disease may occasionally visceralise in immunocompromised hosts [75, 76]. VL involves hematogenous dissemination to the reticuloendothelial system, with heavy parasitization of macrophages in spleen, liver, and lymph nodes producing fever, weight loss, hepatosplenomegaly, pancytopenia, and hypergammaglobulinemia; untreated disease is almost invariably fatal, while a proportion of infections remain asymptomatic or latent and may reactivate years later [74, 77]. A proportion of patients subsequently develop post‐kala‐azar dermal leishmaniasis (PKDL), a dermal complication of VL caused by *L. donovani*, with a latency of months to years that is influenced by the treatment regimen and by host factors such as age [78]. Available drugs for VL and PKDL are associated with substantial toxicity, high cost, and prolonged or parenteral administration, and treatment failure is increasingly reported [77]. PKDL is particularly instructive for the present discussion because it is both a reservoir for transmission and an indication for which the clinical evidence base remains fragmented, with heterogeneous trial designs and endpoints limiting comparison across studies.

Immunologically, VL produces organ‐specific outcomes: progressive dysregulation and loss of architecture in the spleen versus organized granuloma formation and relative parasite control in the liver [79, 80]. This divergence matters here mainly because it means that a delivery system must reach mononuclear phagocytes in two microenvironments with opposite immunological properties, which argues against evaluating candidates in a single organ compartment.

Given the intracellular localization of amastigotes and the therapeutic limitations of conventional drugs, peptide‐based strategies have gained attention as alternatives capable of achieving selective intracellular targeting. Kumar and Chugh [81] demonstrated that the marine peptide tachyplesin exhibits potent antileishmanial activity against *L. donovani*, with an IC_50_ of approximately 4 μM and without inducing ROS. Tachyplesin efficiently internalizes into parasites, disrupts membrane integrity, eliminates intracellular amastigotes at 20 μM, and shows low cytotoxicity toward host cells. The AMP also facilitated plasmid DNA delivery, suggesting dual utility as both a therapeutic and a carrier. Its membrane‐targeting mechanism is particularly relevant given the reduced likelihood of resistance emerging through pathways commonly associated with metabolic drug targets.

**FIGURE 3:**
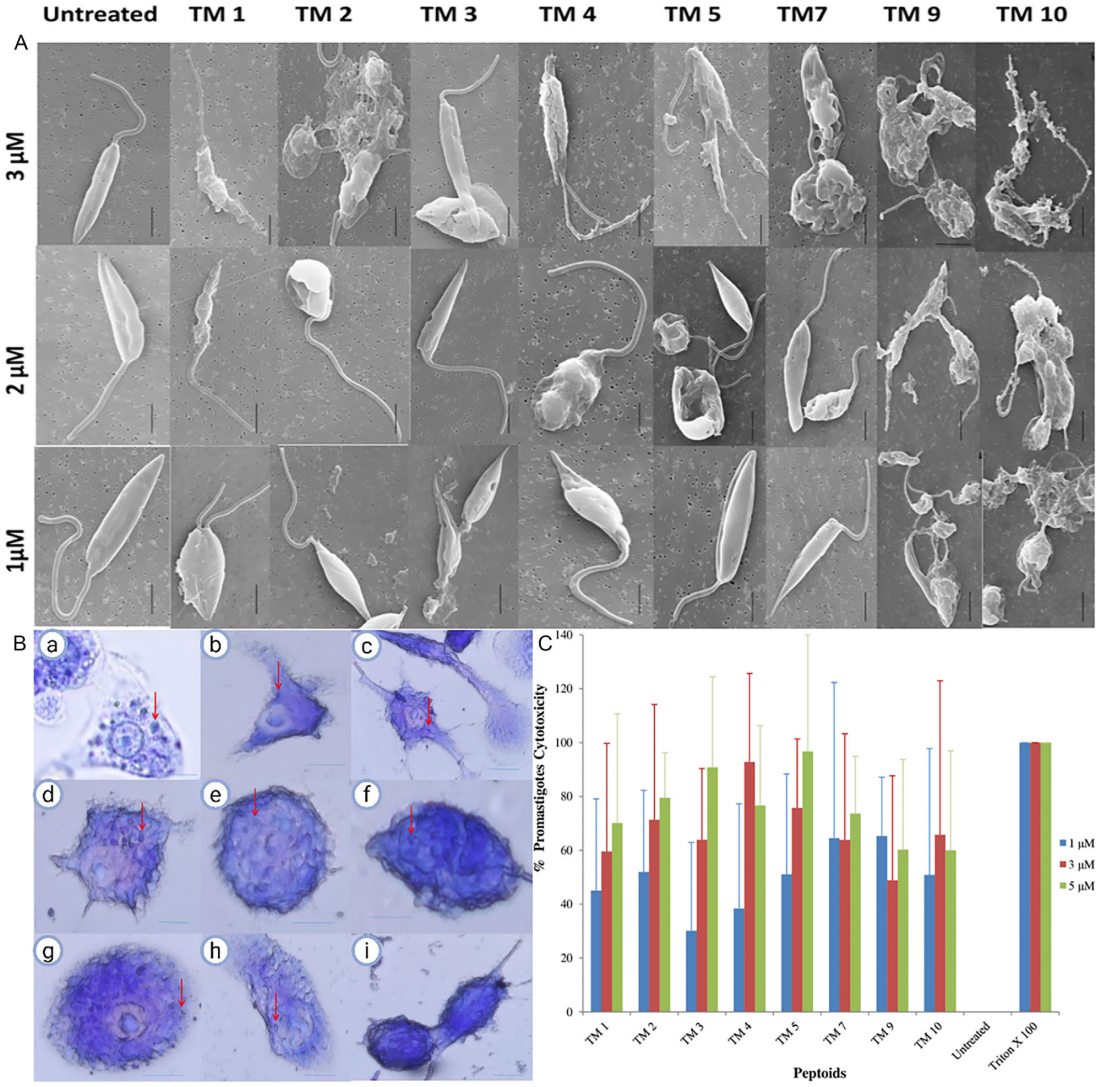
(A) Ultrastructural alterations of *L. donovani* promastigotes after exposure to selected peptoids from the TM1–TM10 series for 4 h. The images in the left column correspond to untreated parasites, and the columns to the right show cells incubated with each peptoid at 1, 2, or 3 µM. Scale bar 1 µm. (B) Giemsa‐stained RAW 264.7 macrophages containing intracellular amastigotes after treatment with the different peptoids at 10 µM. (a) Untreated control, (b) TM1, (c) TM2, (d) TM3, (e) TM4, (f) TM5, (g) TM7, (h) TM9, (i) TM10. Red arrows indicate the amastigote forms of *L. donovani*. Scale bar 10 µm. (C) Cytotoxic activity of the peptoids against *L. donovani* promastigotes measured by LDH release after 4 h of incubation. Reprinted/adapted with permission from [82]. Copyright Wiley 2023.

In a subsequent study, Kumar et al. [82] tested membrane‐acting peptoids transmembrane domains (TM1–TM10) against *L. donovani* in both promastigote and intracellular amastigote forms. Scanning electron microscopy (Figure 3A) showed that a 4 h exposure to 1–3 µM of selected peptoids caused surface roughening, cell shrinkage, and, at higher doses, complete disruption of promastigotes, indicating direct damage to the plasma membrane. Giemsa‐stained RAW 264.7 macrophages infected with *L. donovani* (Figure 3B) displayed fewer and smaller intracellular amastigotes after treatment with 10 µM TM1, TM7, TM9, or TM10 than in untreated controls. Membrane damage was quantified in an LDH release assay (Figure 3C): Promastigotes incubated for 4 h with the peptoids released increasing amounts of LDH with rising concentration, with most compounds reaching more than 60% cytotoxicity at 3 µM and approaching the signal obtained with the Triton X‐100 positive control at 5 µM.

### 3.2 Resistance Mechanisms and Organelle‐Targeted Therapeutic Strategies

Therapeutic failure and relapse in leishmaniasis reflect host factors, treatment limitations, and the genomic plasticity of *Leishmania*. Mosaic aneuploidy, single‐nucleotide variants, copy‐number changes, and extrachromosomal amplification facilitate adaptation under drug pressure [83, 84, 85]. At the functional level, the mechanisms most directly relevant to peptide‐enabled delivery are reduced drug uptake and increased efflux or sequestration [86]. Miltefosine resistance is associated with altered LdMT/ROS3 transporter function and MDR1 activity [87, 88], whereas antimonial resistance involves reduced AQP1‐mediated SbIII entry and ABC‐transporter‐mediated sequestration [89, 90]. These transport‐dependent barriers are retained here because they provide a direct rationale for peptide strategies designed to bypass or modulate drug transport.

Relapse is especially important in immunosuppressed patients and remains substantial in visceral leishmaniasis–HIV coinfection despite recommended therapy [91, 92]. For the present review, the key point is not to catalogue every resistance pathway but to identify mechanisms that peptide systems can plausibly circumvent; the following studies therefore focus on transporter bypass and organelle‐directed activity.

Several studies have investigated peptide‐based approaches to overcome resistance. Luque‐Ortega et al. [93] demonstrated that CPPs can reverse miltefosine resistance in *L. donovani*, a phenotype traditionally linked to defective LdMT transporters. By conjugating a miltefosine analogue to Tat(48–60), the authors restored intracellular drug accumulation and recovered susceptibility in both promastigotes and intracellular amastigotes, even when the transporter was nonfunctional. The conjugate also reduced the parasite burden in infected macrophages and showed activity against *Trypanosoma brucei*, which is intrinsically refractory to miltefosine. This work provides proof of mechanism that CPPs can bypass transport‐dependent resistance and enable activity against strains otherwise unresponsive to treatment. Kabra et al. [94] expanded this concept using computationally designed peptides to counter miltefosine resistance in *L. major*. Through conserved‐motif analysis, structural modeling, and molecular dynamics simulations, they identified peptides—particularly purified fraction 5 (Pg5F) and phytochelatin synthase 2 (PC2)—capable of allosterically modulating transporter proteins involved in resistance. In vitro, these peptides restored sensitivity in resistant promastigotes and amastigotes by increasing drug retention and promoting parasite killing. In vivo, the Pg5F + PC2 combination reduced the parasite burden by 80%–90% even in infections caused by resistant strains. This strategy demonstrates that rationally engineered peptides can overcome transporter‐mediated resistance through allosteric mechanisms that do not depend on the chemical modification of existing drugs.

Beyond transporter‐mediated resistance, peptide therapeutics can also exploit structural vulnerabilities of parasite organelles. One of the most promising targets is the parasite mitochondrion, which in both *Leishmania* and *Plasmodium* exists as a single highly branched organelle with organization and metabolic regulation distinct from that of mammalian cells [95, 96].

In the study by Pitale et al. [97], Halictin‐2—an antimicrobial peptide (AMP) derived from the venom of the eusocial bee *Halictus sexcinctus*—showed potent activity against metacyclic promastigotes and intracellular amastigotes of *Leishmania*. Substitution of serine with threonine at position 5 enhanced α‐helical structure and amphipathicity, improving antiparasitic activity relative to the native peptide. Treated promastigotes exhibited pore formation and membrane disruption. However, intracellular effects were also evident: Exposure to Halictin‐2 induced elevated mitochondrial Ca^2+^ levels, dissipation of mitochondrial membrane potential, decreased ATP production, and increased mitochondrial mass. These alterations triggered bioenergetic collapse and DNA fragmentation, suggesting a mechanism in which membrane destabilization is coupled with mitochondrial dysfunction.

Taken together, the leishmaniasis studies support transporter bypass and parasite organelle disruption as mechanistically plausible peptide strategies, but translation remains constrained by the broader evidence gaps reported for leishmaniasis drug delivery systems and by heterogeneous clinical evidence across disease forms [60, 98]. Candidate evaluation therefore needs to move beyond isolated potency measurements toward intracellular stability, pharmacokinetics, and organ‐specific exposure.

## 4 Antimicrobial Peptides

Having defined the intracellular compartments and diagnostic targets of both parasites, we now turn to the therapeutic entities themselves. AMPs are short polypeptides, typically cationic and amphipathic, that many organisms produce as part of innate immune defenses. Most sequences contain around 10–50 amino acids and fold into α‐helical, β‐sheet, extended, or looped structures that segregate hydrophobic and charged side chains [99]. These architectures support broad activity against bacteria, fungi, and protozoa and are also involved in chemotaxis, cytokine regulation, and tissue repair [100, 101]. From a pharmacological standpoint, AMPs combine rapid killing, micromolar‐to‐submicromolar potency, and a lower tendency to generate classical resistance than many small‐molecule antibiotics [102]. They usually associate microbial membranes through electrostatic attraction to anionic lipids and then insert into the bilayer to form toroidal pores, carpet‐like disruptions, or detergent‐like micellization [103, 104]. Several AMP families also cross membranes and affect nucleic acids, protein‐folding machinery, enzyme networks, or energy metabolism, while shaping host responses in ways that limit excessive inflammation [105].

**FIGURE 4:**
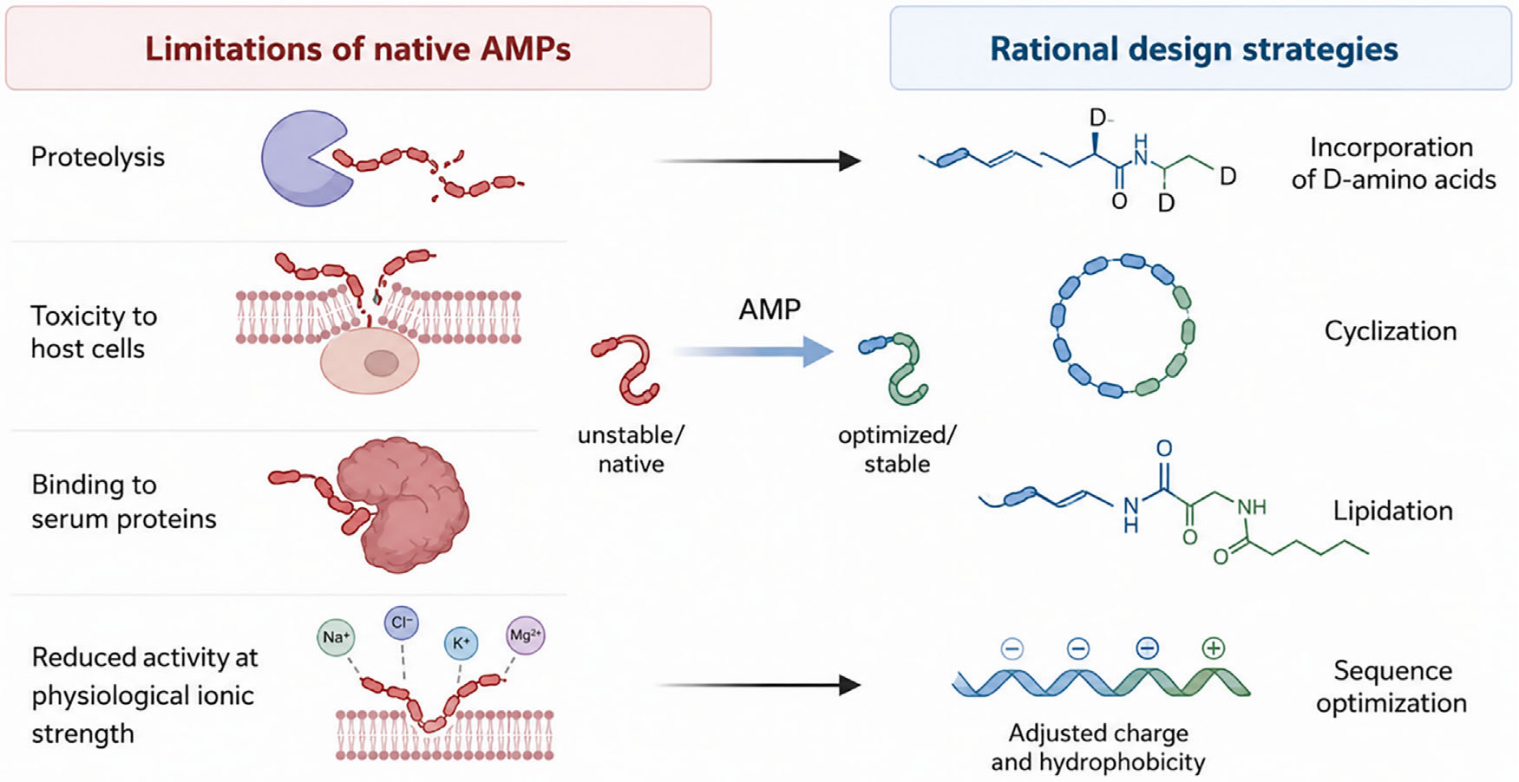
Limitations of native AMPs and rational design strategies for their optimization. Native AMPs exhibit several drawbacks, including susceptibility to proteolysis, toxicity toward host cells, binding to serum proteins, and reduced activity under physiological ionic strength. To overcome these limitations, engineering strategies, such as incorporation of D‐amino acids, peptide cyclization, lipidation, and sequence optimization, are employed, leading to improved stability, selectivity, and biological activity of AMPs.

Native AMPs nevertheless face hurdles when developed as drugs, including fast proteolysis, reduced activity at physiological ionic strength, binding to serum proteins, and toxicity toward host cells [106]. Rational design strategies mitigate these limitations by trimming or reorganizing sequences, adjusting net charge and hydrophobicity, introducing D‐amino acids or nonnatural residues, imposing cyclic constraints, or attaching lipid moieties [107] (Figure 4). These modifications can prolong half‐life, bias interactions toward pathogen membranes, reduce hemolysis, and facilitate large‐scale synthesis and quality control [108, 109]. Formulation in nanoscale carriers offers an additional means to optimize AMP performance [110]. Encapsulation or adsorption in liposomes, polymeric nanoparticles, dendritic nanogels, mesoporous silica, or metallic nanostructures can protect peptides from proteases, modulate release, and favor accumulation at infected sites. By tuning surface charge, adding targeting ligands, or incorporating stimuli‐responsive components, these carriers gain better access to biofilms and intracellular compartments and can be integrated into combination regimens with existing drugs [111, 112].

The physicochemical traits that support antibacterial activity also underpin the activity against protozoan parasites. Recent analyses describe how cationic peptides can destabilize parasite membranes, disturb calcium balance, collapse mitochondrial potential, or interfere with key metabolic pathways in *Plasmodium* and *Leishmania* while maintaining tolerable toxicity in mammalian cells [113]. Particular attention has been given to AMPs with immunomodulatory or cell‐penetrating properties, since they can influence host–parasite interactions and assist intracellular delivery of therapeutic cargos [114]. Experimental evidence indicates that both animal‐derived and synthetic AMPs can act at multiple points of protozoan life cycles. Frog skin peptides and related analogues have shown activity against *P. falciparum* blood stages and *Leishmania mexicana* and in some cases also against *Trypanosoma cruzi*, suggesting that broad antiparasitic profiles are achievable [115]. In leishmaniasis, cell‐penetrating and lytic peptides are being developed to reach amastigotes within macrophage phagolysosomes, either as stand‐alone agents or as carriers for nucleic acids and small‐molecule drugs [116]. When incorporated into nanocarriers, these peptides may gain proteolytic protection, improved pharmacokinetics, and higher intracellular accumulation, thereby increasing their translational relevance [117]. In this review, AMPs and their nanoformulations are treated as adjustable tools whose sequence and carrier can be aligned with the biological constraints of each protozoan infection. The following sections examine how these concepts have been applied to malaria and leishmaniasis, with emphasis on hepatic and erythrocytic stages of *Plasmodium* and on *Leishmania* amastigotes residing in macrophages.

## 5 Peptide‐Based Nanocarrier Systems for Drug Delivery in Leishmaniasis and Malaria

Interest in peptide‐based nanocarriers for malaria arises from persistent therapeutic limitations. Parasite resistance, poor solubility of several antimalarials, and systemic toxicity continue to reduce the real effectiveness of conventional formulations [118, 119]. In leishmaniasis, the need is equally pressing as available chemotherapy remains associated with toxicity, parenteral administration, and resistance, even when drugs retain intrinsic activity against the parasite [120]. These two contexts, however, should not be treated as equivalent. In leishmaniasis, the immediate therapeutic target is usually the infected macrophage and its parasitophorous vacuole, an acidic and proteolytic niche that demands precise intracellular trafficking decisions [121]. In malaria, by contrast, the current landscape includes liposomes, polymeric nanoparticles, dendrimers, micelles, niosomes, lipid systems, and exosomes. This diversity suggests a focus on reshaping pharmacokinetics and biodistribution rather than overcoming a single subcellular barrier [122].

For this reason, in leishmaniasis the advantages of nanoformulation are mainly linked to macrophage uptake, improved therapeutic index, and reduced toxicity. In malaria, greater weight is given to solubility, release control, and systemic exposure [123]. Within this framework, peptides are valued not as decorative elements but as engineering components. Their structural flexibility and biocompatibility allow them to function as surface ligands, self‐assembling elements, or drug conjugation units [124]. The literature on peptide‐functionalized nanomedicine shows that this peptide layer can be integrated into lipid, polymeric, metallic, or silica cores and modify the biological interface of the carrier without replacing its core architecture [125, 126]. At the same time, studies on therapeutic peptides point out that proteolytic degradation, short half‐life, and formulation instability remain major barriers to clinical use [127].

When CPPs are used, their advantage goes beyond facilitating cellular entry. These sequences can also help overcome barriers such as mucus, immune clearance, and nonspecific accumulation, provided that the relationship between the sequence, charge, and nanocarrier is carefully tuned [128, 129]. Internalization, however, is only the first step. Many nanocarriers fail later during intracellular trafficking, resistance evasion, or cargo release in the correct compartment [30]. This limitation is particularly evident in systems that rely on endocytosis, where endosomal escape remains a major bottleneck even for more mature platforms such as lipid nanoparticles [130]. The recent shift toward organelle‐targeted nanoparticles reflects an attempt to recover an effective dose at the level where intracellular target exposure is determined [131].

At this stage, peptide‐ or lipopeptide‐guided inorganic nanoparticles offer a useful toolkit. They combine the surface and physical properties of the core with specific recognition and stimulus responsiveness provided by the peptide [132]. A similar logic appears in peptide‐based skin formulations, where the peptide can simultaneously contribute to barrier penetration, bioactivity, and local retention. This combination is especially relevant for cutaneous leishmaniasis [133]. The association between peptides and polymers introduces an additional level of control. Co‐assembled architectures can improve stability, bioavailability, and responsiveness without always requiring more complex covalent synthesis [134]. Even in liposomal systems designed for other indications, peptides derived from phagocytosis signals have shown that a well‐chosen ligand can redirect cellular uptake and alter the pharmacology of the same carrier [135].

This functional flexibility should not obscure the fact that peptide modification also alters the nanoarchitecture. Changes in ultrastructural organization, surface adsorption, and cellular uptake have been observed in liposomes decorated with tissue affinity peptide sequences [136]. The use of stable peptide ligands in D configuration shows that improvement does not always come from adding new functions but from preserving stability, reducing self‐aggregation, and maintaining a surface compatible with prolonged circulation [137, 138]. Rational engineering of polymeric nanoparticles for biologics follows a similar direction, protecting fragile cargos, controlling release, and increasingly relying on predictive tools for future translation [139]. Carrier morphology remains an underestimated variable. Studies with sugar‐based nanoparticles loaded with peptides have shown that shape and size influence cellular uptake and the duration of pharmacological effects [140].

**FIGURE 5:**
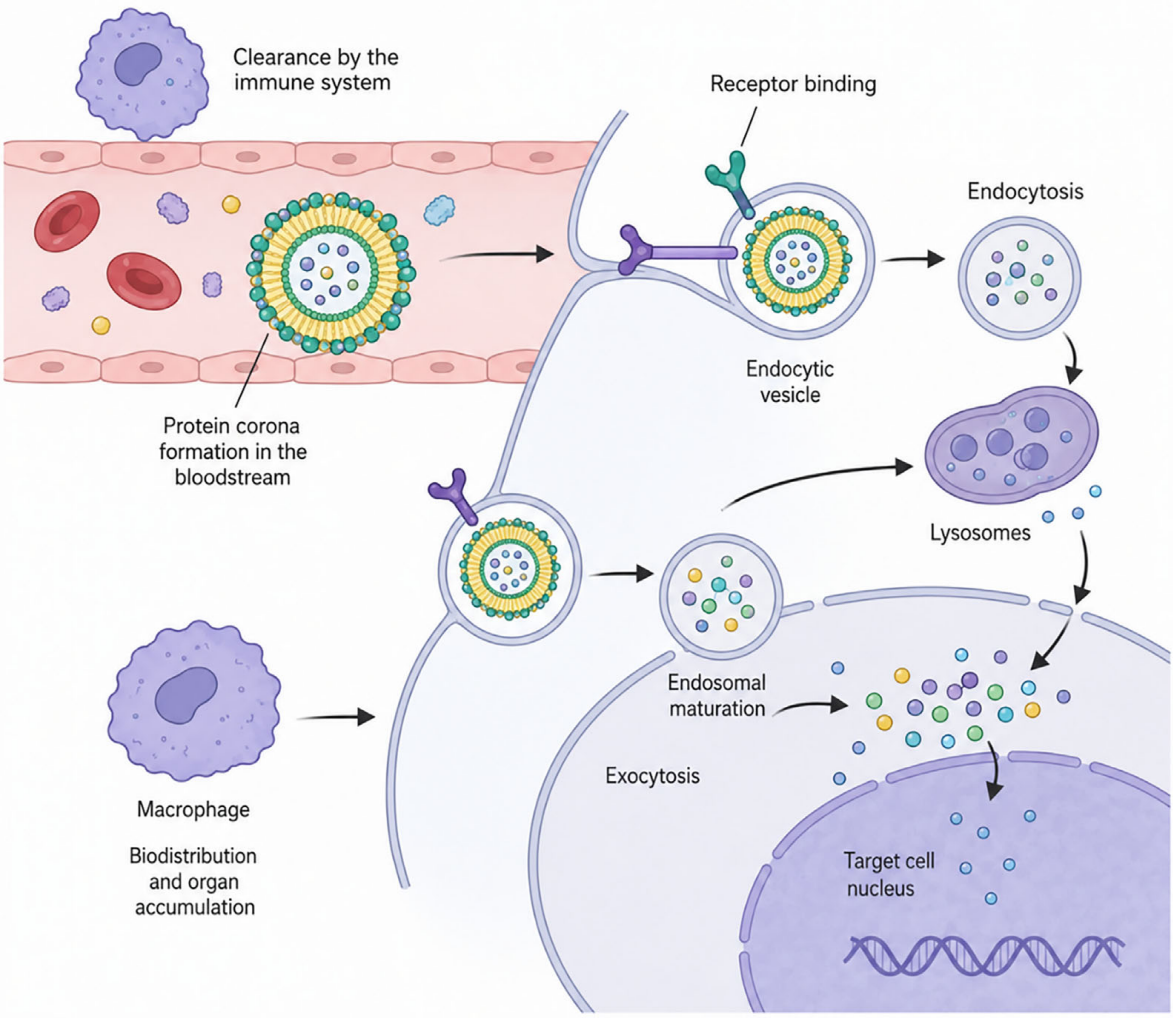
Schematic overview of the in vivo fate and intracellular trafficking of drug‐loaded nanoparticles after systemic administration, illustrating protein corona formation, interaction with the MPS, tissue distribution, receptor‐mediated cellular uptake, endosomal maturation, and intracellular drug release pathways.

Clinical translation depends on more than nanoparticle design. The difficulty lies in bridging design, tissue exposure, preclinical reproducibility, safety, manufacturing, regulation, and business considerations, as outlined in the DELIVER framework [141]. Influential perspectives in the field converge on this view and place the barriers of nanomedicine across physiological, immunological, clinical, and regulatory domains. Increased uptake alone cannot be taken as evidence of therapeutic value [142]. Manufacturing remains a particularly difficult aspect. Many formulations perform well at the analytical scale but fail to produce homogeneous batches for large animals or clinical trials, which explains the growing interest in controlled continuous microfluidic synthesis [143, 144]. Characterization in real biological media is equally critical, since the protein corona reshapes nanocarrier identity and influences pharmacokinetics, biodistribution, and efficacy [145]. This sequence of biological interactions, from corona formation in the bloodstream to intracellular trafficking, processing, and clearance, is shown in Figure 5.

Current discussions extend beyond the traditional concept of the protein corona. The idea of a biocorona includes proteins, lipids, and other biomolecules that dynamically shape nanoparticle interactions with the organism [146]. Recent integrated analyses confirm this complexity, showing that size, zeta potential, material, and incubation time drive corona composition toward patterns associated either with receptor‐mediated uptake or with immune recognition and rapid clearance [147, 148]. This adsorbed layer influences both nanoparticle distribution and cargo release, as demonstrated in PLGA systems, where the protein corona modifies drug release profiles [149]. Uptake by the mononuclear phagocyte system (MPS) adds another level of constraint. Its dominance has even led to strategies aimed at temporary blockades to extend circulation and improve delivery [150].

One possible response is to redesign the surface to induce less immunogenic coronas. Glycopolymeric nanoparticles have shown reduced adsorption of immunoglobulins and complement, lower macrophage uptake, and improved distribution compared to PEGylated counterparts [151, 152]. Another approach is cell membrane biomimicry, which seeks to transfer immune evasion, longer circulation, and dynamic targeting through a biological coating [153]. Current evidence indicates that large‐scale production, standardization, and long‐term safety assessment remain unresolved challenges [154, 155]. Evaluation models also remain a source of systematic error. Limited translation of nanomedicine is partly due to preclinical systems that fail to reproduce relevant biological interactions, which is why organoids are gaining attention as a more demanding filter [156].

**FIGURE 6:**
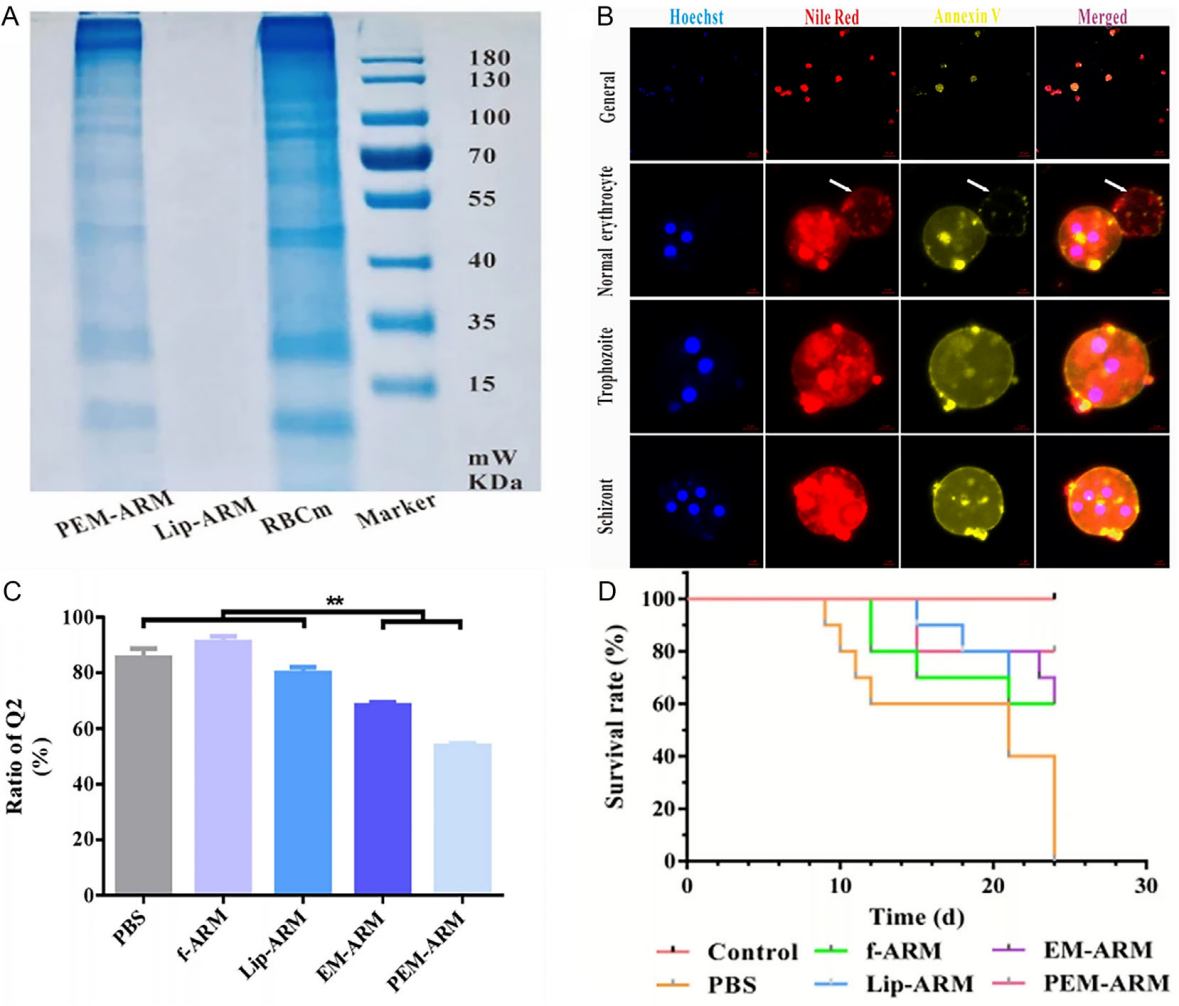
CLIPPKF‐modified erythrocyte membrane nanoliposomes for targeted artemether delivery in experimental malaria. (A) SDS‐PAGE profiles of red‐blood‐cell membranes (RBCm), artemether liposomes without membrane coating (Lip‐ARM), and CLIPPKF‐modified erythrocyte membrane‐camouflaged artemether liposomes (PEM‐ARM), showing that PEM‐ARM preserves the characteristic RBCm protein pattern. (B) Confocal fluorescence images of normal erythrocytes and *P. berghei* ANKA (pbANKA)‐infected red blood cells at ring, trophozoite, and schizont stages after incubation with Nile‐Red‐labeled PEM‐ARM. Nuclei (Hoechst 33,342) are shown in blue, nanoparticles (Nile Red) in red, and externalized phosphatidylserine (Annexin V–Cy5) in yellow, with preferential association of PEM‐ARM to infected cells. (C) Inhibition of merozoite invasion, expressed as the percentage of double‐positive erythrocytes in quadrant Q2 after co‐incubation of Hoechst‐labeled merozoites with CFDA‐SE‐labeled red blood cells in the presence of PBS, free artemether (f‐ARM), Lip‐ARM, erythrocyte membrane‐camouflaged artemether liposomes (EM‐ARM), or PEM‐ARM. Lower Q2 values correspond to stronger blockade of invasion. (D) Kaplan–Meier survival curves of pbANKA‐infected C57BL/6 mice treated intravenously with PBS, f‐ARM, Lip‐ARM, EM‐ARM, or PEM‐ARM at an equivalent artemether dose of 2.5 mg kg^−1^, showing the survival benefit achieved with the CLIPPKF‐targeted formulation. Reprinted/adapted with permission from He et al. [32]. Copyright Springer Nature 2023.

He et al. [32] provided a particularly instructive demonstration in malaria by combining the erythrocyte membrane, artemether, and the peptide CLIPPKF. This system captured merozoites and increased drug accumulation in infected erythrocytes, reducing inflammation, anemia, and mortality in mice infected with *P. berghei* (Figure 6). Despite its value, broader challenges remain. Work on macrophage membrane‐coated nanoparticles and other biomimetic systems highlights ongoing issues with extraction reproducibility, batch consistency, and long‐term biosafety [157]. Kumar et al. [158] proposed a more compelling approach for leishmaniasis by conjugating amphotericin B with an IL‐10 antagonist peptide and encapsulating the system in gamma cyclodextrin. The resulting nanoparticles, around 40 nm in size, reduced parasite burden and improved immune and safety profiles compared to the free drug. This strategy aligns with the biology of infection, as macrophage‐targeted designs are valued for combining biocompatibility, targeting specificity, and immunomodulation, attributes that are difficult to separate in antileishmanial therapy [159].

Wang et al. [160] formulated amphotericin B nanocrystals in dissolving intradermal microarrays. These showed greater lymphatic accumulation and lower deposition in toxicity‐related organs compared to intravenous AmBisome. Recent clinical trials with liposomal amphotericin B in PKDL provide a clinical benchmark for these formulations. New platforms will need to demonstrate clear advantages over shorter and more practical regimens, not only over the free drug [161]. Cosco et al. [162] reinforced this point by showing that reformulation of meglumine antimoniate in PLA nanocapsules increased plasma half‐life, reduced renal accumulation, and decreased parasite burden. This suggests that translation may depend more on refining robust technologies than on endlessly increasing surface complexity. In malaria, a recent systematic analysis of nanotherapeutics still describes a field dominated by experimental models and the ongoing challenge of developing strategies that are both specific and cost‐efficient [163].

In this context, studies on corona formation in gold nanoparticles show that baseline surface chemistry can either promote or nearly eliminate nonspecific adsorption. A targeting peptide should therefore not be added as a final functional label but designed together with the coating that will define its actual identity in blood [164]. Taken together, peptide‐based nanocarriers for malaria and leishmaniasis will have a realistic path toward clinical application only when the peptide addresses a defined biological barrier, the nanocarrier maintains a reproducible identity in real fluids, and the formulation is integrated into a complete pharmaceutical strategy that brings together chemistry, delivery platforms, and manufacturing [165]. A quantitative comparison of representative peptide‐enabled nanoplatforms for malaria and leishmaniasis is shown in Table 1. While these advances highlight the translational challenges of nanotherapeutics, similar design principles are now being leveraged in diagnostic systems.

**TABLE 1:** Quantitative comparison of peptide‐enabled nanoplatforms investigated for malaria and leishmaniasis.

Disease/model	Peptide component	Nanoplatform	Key antiparasitic efficacy	Toxicity/safety	Stage	Refs.
Malaria*—P. berghei* K173, C57BL/6J mice	cRGD, M‐cell targeting	cRGD/PDA‐modified nanoliposomes carrying DSSC	>80% *P* *lasmodium* inhibition at 27.4–219.4 μmol/kg	Caco‐2 viability >80%	Preclinical, in vivo	[166]
Malaria*—P. berghei* ANKA, C57BL/6 mice	CLIPPKF, PS targeting	RBC membrane‐camouflaged artemether nanoliposomes (PEM‐ARM)	Merozoite invasion 53.80% versus 85.37% control; 2.5 mg/kg prolonged survival	Hemolysis <5%	Preclinical, in vitro/in vivo	[32]
Leishmaniasis*—L. major* promastigotes/amastigotes	B84 lipopeptide fraction containing surfactin and bacillomycin D	Chitosan nanoparticles	IC_50_ 14.37 μg/mL promastigotes; 22.45 μg/mL amastigotes	HC_50_ 770 μg/mL; RAW264.7 LC_50_ 234.56 μg/mL	Preclinical, in vitro	[167]
Leishmaniasis—intracellular *L. major*	MMP‐2/9‐activatable melittin	Liposomes and albumin nanoparticles	~80% reduction at 25 μg/mL; 7.0 ± 1.5 versus 35.0 ± 3.2 amastigotes/macrophage	Formulations reported as nontoxic at 25 μg/mL	Preclinical, in vitro	[168]
Leishmaniasis—experimental infection	IL‐10 antagonist peptide conjugated to AmB	γ‐Cyclodextrin nanoparticles (~40 nm)	Significant reduction in parasite burden and increased amastigote clearance; quantitative % NR	Improved hematological and biochemical safety versus AmB	Preclinical, in vitro/in vivo	[158]
Cutaneous leishmaniasis*—L. major*, mouse	PADRE	PADRE‐derivatized dendrimer complexed with liposomal AmB (PDD/LAmB)	Drug efficacy increased 83%; APC targeting increased tenfold	Reduced toxicity versus LAmB treatment	Preclinical, in vivo	[169]

Abbreviations: AmB, amphotericin B; DSSC, disulfide‐modified dihydroartemisinin prodrug; HC_50_, 50% hemolytic concentration; LC_50_, 50% lethal concentration; NR, not reported; PS, phosphatidylserine.

## 6 Aptamer‐ and Peptide‐Driven Nanodiagnostics

Early and accurate identification of malaria and leishmaniasis cases remains essential for effective disease control [170]. Despite their utility in certain contexts, conventional diagnostic procedures still present multiple drawbacks that restrict early case recognition and sustained parasite management [171]. In malaria, microscopic examination of blood smears continues to be regarded as the reference method, though it requires trained personnel and often misses infections of low parasitemia or mixed *Plasmodium* species [172]. In leishmaniasis, serological methods, such as enzyme‐linked immunosorbent assay (ELISA), indirect fluorescent antibody test, and rK39‐based rapid tests, often give false‐positive reactions because of antibody cross‐recognition of *T. cruzi* and other co‐endemic pathogens, which reduces diagnostic reliability [173]. Routine use is further restricted by the requirement for centralized laboratories, poor stability of many reagents at high ambient temperatures, and limited suitability for use directly in the field [174].

Over the last decade, nanotechnology has been combined with molecular recognition elements to broaden the diagnostic toolbox. Gold nanoparticles, quantum dots, carbon nanotubes, graphene‐derived structures, and metal–organic frameworks are increasingly coupled to aptamers or synthetic peptides to generate sensing platforms capable of detecting target molecules at pico‐ to femtomolar levels [175, 176]. When properly optimized, these devices can satisfy the analytical and operational criteria defined in the STARD and REASSURED guidelines, including precision, robustness, cost control, and applicability in decentralized health services [177, 178]. Translation to clinical practice, however, still depends on rigorous validation in representative patient cohorts, reproducible large‐scale manufacturing, and integration with existing diagnostic workflows and distribution channels [179, 180]. In endemic and resource‐limited settings, the appeal of peptide‐ and aptamer‐based nanosensors lies in their analytical sensitivity, chemical stability, modular synthesis, and greater compatibility with scalable manufacturing and decentralized deployment compared with conventional antibody‐based formats.

### 6.1 Malaria: From HRP2 Deletions to Multiplexed Panels

A persistent challenge in malaria diagnosis is keeping assay sensitivity when parasite genotypes change. The rising prevalence of pfhrp2 and pfhrp3 deletions in several regions of Africa and South America has undermined the performance of tests that rely exclusively on HRP2 detection [181]. In response, laboratories and control programs are moving toward multiplex formats that include additional antigens such as PfLDH and PfGDH, which support species discrimination and help maintain diagnostic yield where HRP2‐based assays have become unreliable.

Nanomaterials have enabled more sensitive detection formats for malaria biomarkers. Electrochemical and optical devices built with gold nanoparticles, quantum dots, or silicon nanowires can quantify PfHRP2 and pLDH at picogram‐per‐milliliter levels, a range not typically reached by routine rapid diagnostic tests [182, 183]. These systems have been configured for electrochemical readouts, fluorescence, or surface plasmon resonance, which facilitates the detection of low‐parasitemia infections and early‐stage disease. Their operation with portable instrumentation supports their use in places that do not have access to centralized laboratory facilities [184].

**FIGURE 7:**
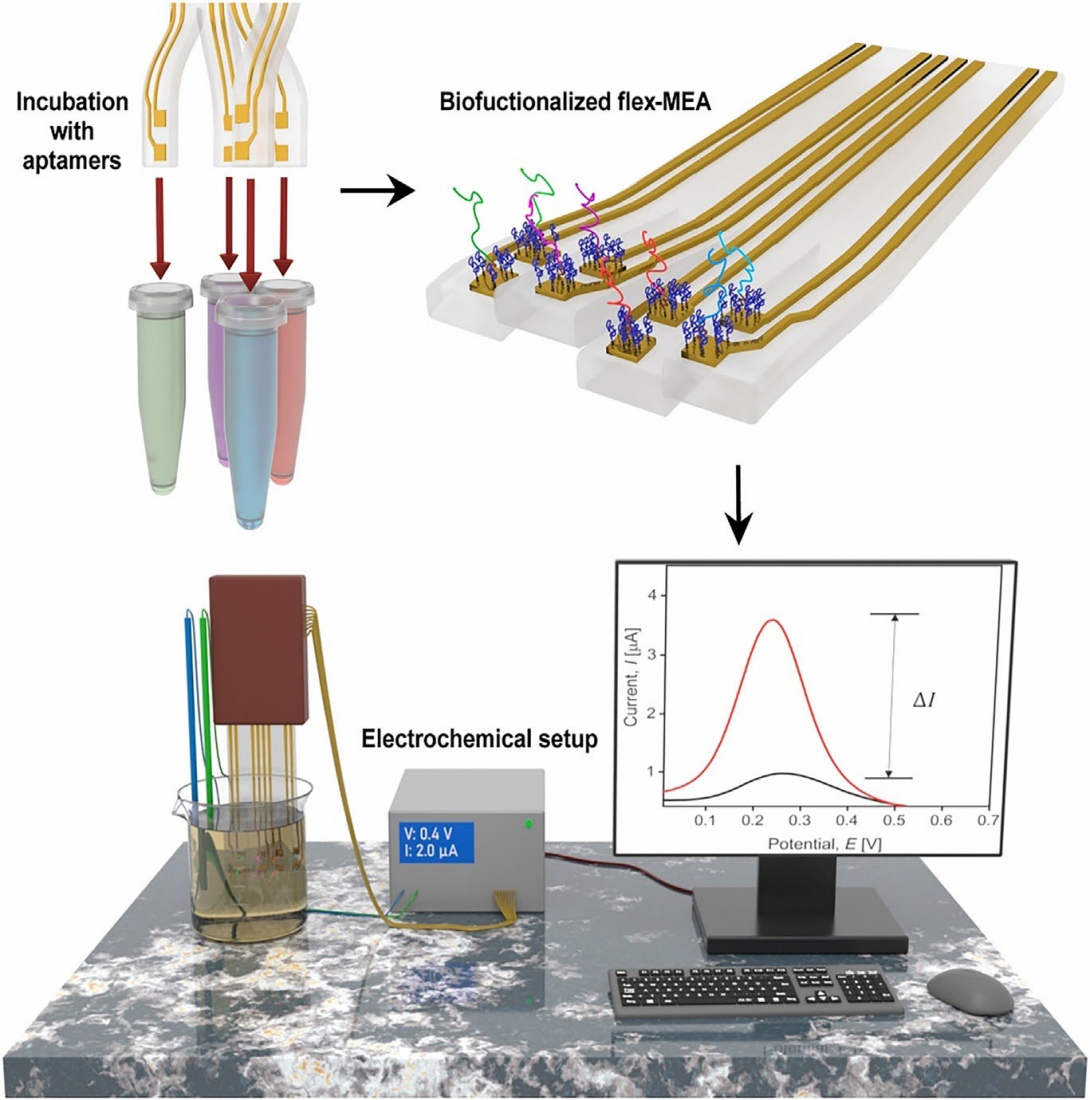
Multi‐target functionalization of the flex‐MEA and detection of the corresponding analytes. The flex‐MEA is modified with four aptamers, 2008s (green), pL1 (purple), LDHp11 (red), and 2106s (blue), and connected to the electrochemical cell used for biosensor measurements. The plot shows the change in peak current between differential pulse voltammograms recorded before and after target detection. Reprinted/adapted with permission from [185]. Copyright Elsevier 2021.

Figueroa‐Miranda et al. [185] developed an aptamer‐based electrochemical platform using a flexible multielectrode array (flex‐MEA) functionalized with four aptamers to detect PfLDH, PvLDH, and PfHRP2 in parallel (Figure 7). The device achieved sensitivity above 75% at parasitemias near 0.001% (50 parasites/µL), in line with or exceeding performance criteria set by the WHO. Printed electrodes with nanostructured surfaces increased the number of available sites for aptamer attachment and improved electron transfer, which allowed several targets to be evaluated simultaneously.

Similarly, Minopoli et al. [186] presented a plasmonic detection system that identified PfLDH directly in whole blood without preprocessing. Their design combined surface‐immobilized antibodies with fluorescent aptamers in a sandwich configuration and reached detection limits below 1 pg/mL (<30 fM). The use of ordered AuNP arrays prepared by block copolymer micellolithography enhanced plasmonic coupling, and controlled antibody orientation improved access to the relevant epitopes. Complementary to antibody‐ and aptamer‐based detection, Hoang et al. [33] introduced a peptide‐driven fluorescent method targeting merozoite surface protein 2 (MSP2) of *P. falciparum*. Two peptide candidates (M2.9 and M2.17), selected through computational epitope screening, were immobilized on immunochromatographic strips and coupled to europium nanoparticles using EDC/NHS chemistry. The assay detected ten parasites/µL in FLISA and five parasites/µL in fluorescent immunochromatography for the 3D7 strain, representing a marked improvement over conventional rapid diagnostic tests. The strong and stable luminescence of europium nanoparticles, together with the thermal stability of the designed peptides, contributed to the system's performance.

### 6.2 Leishmaniasis: Specificity Against Coinfections

In leishmaniasis, the main diagnostic difficulty is achieving specificity in areas where several pathogens circulate simultaneously [187]. Serological tests often cross‐react with *T. cruzi* and related organisms, generating false positives that complicate case confirmation and epidemiological reporting [188]. This has encouraged the use of nanosensors based on defined molecular targets, synthetic peptides, or aptamers instead of whole‐parasite antigens, which reduces unwanted interactions and improves analytical precision.

Current work has focused on markers such as GP63, KMP‐11, and kinetoplast DNA (kDNA) [189]. Quantum‐dot platforms carrying DNA probes or aptamers, together with graphene‐based sensors, have achieved better discrimination and less interference than conventional serology [190]. These systems also allow the use of peripheral blood or other minimally invasive samples, avoiding procedures such as bone marrow or splenic aspiration. Can et al. [191] reported a simple colorimetric assay using gold nanoparticles coated with KMP‐11 to detect antibodies against GP63. Antigen–antibody binding induced nanoparticle aggregation, visible as a red‐to‐purple color change and quantifiable by UV–vis spectroscopy. The method detected antibody dilutions up to 1:640 with recovery values around 95%–100%, supported by a stable protein corona that amplified the plasmonic response. Perk et al. [192] extended this concept by building an electrochemical sensor based on copper metal–organic frameworks modified with KMP‐11 to capture anti‐KMP‐11 antibodies. The porous MOF matrix provided a large surface for antigen immobilization via EDC/NHS chemistry, enabling detection of dilutions up to 1:1500 with a relative standard deviation of 4.67% in assays using crude antigens and rabbit sera. Peptide‐based architectures add further flexibility. Braz et al. [34] developed a disposable electrochemical sensor that combined solid‐binding peptides with carbon nanomaterials. Two variants (395‐KKG and 395‐G) were engineered with different anchoring domains to interact selectively with graphene oxide or graphene quantum dots. The 395‐G construct on graphene quantum dots showed the best performance, distinguishing positive from negative leishmaniasis sera with 95% confidence.

Aptamers are gaining importance as recognition elements in parasitic diagnostics [193]. In malaria, aptasensors directed against PfHRP2 and pLDH have shown greater thermal robustness and sensitivity than ELISA [194]. Comparable progress has been reported in leishmaniasis, where aptamers against GP63 and other surface antigens have been incorporated into fluorescent and plasmonic formats with high target specificity [195]. Their chemical stability and reproducible synthesis support their development for wider use [196].

Synthetic peptides are also being explored because of their modular structure and predictable behavior. Several reports describe selective binding to *Leishmania* and *Plasmodium* proteins using peptide‐based optical or electrochemical sensors [197, 198]. In many of these designs, a single peptide scaffold can be adapted for both detection and antiparasitic activity, opening the door to theranostic formats in which diagnostic and therapeutic functions are combined in the same molecular system [199]. Aptamer‐ and peptide‐based nanosensors therefore provide a path toward more specific and scalable diagnostic tools for leishmaniasis, provided that manufacturing parameters, environmental stability, and validation in different endemic settings are addressed.

### 6.3 Implementation Standards and Field Validation

Although nanodiagnostic platforms have advanced considerably, their incorporation into routine workflows requires compliance with international validation standards and alignment with national surveillance strategies. The STARD and REASSURED frameworks remain the main reference points. STARD focuses on transparent reporting of analytical and clinical performance, including sensitivity, specificity, ROC curves, the Youden index, justification for sample size, and the suitability of the biological matrix used [178]. REASSURED specifies the operational attributes needed for point‐of‐care use such as affordability, accuracy, short turnaround times, ease of use, robustness, minimal equipment requirements, and digital compatibility, all of which determine whether a test can be adopted at scale [177].

Validation must consider matrices encountered in real clinical settings. In malaria, capillary blood from finger‐prick sampling is preferred, with target readout times of 20 min or less [200]. For cutaneous leishmaniasis, swabs collected directly from lesions give access to parasite biomarkers but often require antifouling treatments and optimized blocking steps to limit matrix interference. In visceral disease, serum and urine function as practical alternatives, provided that protein interactions and nonspecific adsorption are addressed [201].

Thermal stability is a point of clear divergence between aptamer‐ or peptide‐based systems and antibody‐based assays. Antibodies of approximately 150 kDa are vulnerable to loss of structure at room temperature or 37 °C, creating dependence on refrigerated transport and storage in low‐resource environments [202]. Aptamers can be subjected to repeated denaturation–renaturation cycles, preserve their binding capacity during long storage at room temperature, and tolerate heating up to about 95 °C without a measurable loss of function [203]. Peptides, although more vulnerable to proteolytic degradation, are inexpensive to synthesize and can be chemically modified to increase conformational robustness, so that their activity is retained after immobilization or covalent conjugation [204, 205].

Economic evaluations indicate a favorable cost profile for these platforms. Electrochemical aptasensors can achieve per‐test prices close to one US dollar when coupled to reusable readers, whereas many commercial rapid tests fall in the 2.54–2.83 USD range [203]. Fluorescent peptide strips directed to merozoite surface protein 2 reach detection limits up to 20‐fold lower than standard malaria RDTs, which makes them attractive candidates for decentralized screening programs [33]. Device architecture is also compatible with large‐scale use; immunosensors based on metal–organic frameworks can be fabricated by straightforward deposition onto screen‐printed electrodes, supporting scalable production [206]. Modular assay panels that combine multiple biomarkers additionally permit adjustment to pfhrp2/3 deletions and to regional differences in transmission patterns [207].

Quality assurance is a necessary component of implementation. In a large multiplex malaria antigen detection study, positive control curve coefficients of variation remained below 5% throughout the survey except for HRP2 in the fifth month (11%), and 160,641 of 161,088 potential sample–antigen data points (99.7%) passed quality checks [208]. These data emphasize the need to coordinate analytical performance with reproducible manufacturing and rigorous field validation to ensure suitability for endemic settings.

## 7 Translational Barriers: Why Few Peptide Candidates Reach Clinical Use

The preceding sections describe a large and mechanistically diverse set of peptide‐based candidates for malaria and leishmaniasis. Set against this activity, the clinical output is small. Curated compilations of regulatory approvals list 894 FDA‐approved therapeutic proteins, of which only 85 are peptides or polypeptides, and essentially none targets a protozoan infection [209]. For AMPs specifically, four decades of development have yielded very few marketed products, with attrition attributed principally to instability and toxicity rather than to lack of potency [210]. The pattern of late‐stage failure is instructive: Topical omiganan in a phase 2 randomized controlled trial in atopic dermatitis reduced *Staphylococcus aureus* abundance by 93.5% and restored microbiome diversity yet produced no significant clinical improvement [211], illustrating how a validated mechanism can fail to translate into clinical benefit. Similarly, murepavadin, the most clinically advanced target‐specific peptide antibiotic, selects for resistance through lipopolysaccharide modification, showing that the low‐resistance assumption often invoked for peptides does not hold universally [212]. The following subsections analyze the specific barriers that generate this attrition and, where possible, the mitigation strategies supported by experimental evidence. Table 2 summarizes this analysis.

**TABLE 2:** Translational barriers for peptide‐enabled nanoplatforms in malaria and leishmaniasis, their specific manifestation in antiparasitic candidates, and evidence‐supported mitigation strategies.

Translational barrier	Translational implication	Priority strategies	Refs.
Proteolytic and metabolic instability	Loss of active peptide before reaching the parasite or intracellular target	Cyclization, nonnatural residues, terminal modification, lipidation	[213]
Limited systemic exposure and bioavailability	Short circulation time and restricted access to infected tissues	Half‐life extension, carrier‐assisted delivery, permeability enhancement	[165]
Protein corona and immune recognition	Altered biodistribution, targeting loss, and accelerated nanocarrier clearance	Surface chemistry optimization and biologically relevant corona profiling	[152]
Manufacturing complexity	High material consumption, difficult scale‐up, and increased cost of goods	Process intensification, reduced solvent synthesis, and simplified constructs	[214]
Regulatory and analytical burden	Peptide‐related impurities and structural heterogeneity complicate quality control	Orthogonal characterization, impurity profiling, and early CMC integration	[215]
Limited deployability in endemic settings	Complex administration, cost, and treatment burden can prevent real‐world adoption	Patient‐oriented target product profiles, simple dosing, and affordable formulations	[216]
Suboptimal candidate selection	Potent sequences may fail because stability, toxicity, and developability are considered too late	Multiparametric screening and AI‐assisted sequence optimization	[217]

### 7.1 Proteolytic Instability and Metabolic Clearance

Susceptibility to proteolysis is the most frequently cited cause of failure in peptide development, and it operates at every level from the injection site to the intracellular compartment [218]. The problem is compounded in antiparasitic applications because the relevant matrices, including plasma, the macrophage phagolysosome, and the parasitophorous vacuole, are all proteolytically aggressive. Chemical solutions are well established. D‐amino acid substitution, backbone cyclization, and unnatural residues raise protease resistance, while side‐chain stapling additionally pre‐organizes the α‐helix, improving both membrane permeability and proteolytic stability relative to the linear parent [219, 220]. Glycosylation and lipidation modulate pharmacokinetics while preserving antimicrobial activity, and self‐assembly can be exploited directly: Rationally designed self‐assembling peptide nanofibers retain antimicrobial activity after protease exposure that inactivates the corresponding monomers [221, 222]. The critical observation for this field is methodological rather than chemical. Cytotoxicity and activity measured in serum‐free media diverge substantially from values obtained in the presence of serum, so that selectivity indices reported without serum systematically overestimate the therapeutic window [223]. For antiparasitic peptide studies, IC_50_ values obtained under serum‐free or low‐serum conditions and without measured plasma half‐life should therefore be interpreted cautiously, because such conditions can overestimate the therapeutic window. This is a major correctable weakness when it occurs.

### 7.2 Pharmacokinetics and Route of Administration

Unmodified peptides are cleared within minutes by glomerular filtration and peptidase activity. Organ impairment can further alter their clearance. A regulatory review of approved products found that renal impairment effects were documented in the labeling of 57% of peptides, with dose adjustment required in 37% of those cases [224]. This is a material concern in visceral leishmaniasis and severe malaria, where renal and hepatic dysfunction are common features of the disease itself. Half‐life extension is technically feasible—Fc conjugation supports prolonged systemic exposure while accommodating nonnatural residues, and lipidation designed to promote albumin binding or membrane anchoring markedly prolongs activity in vivo—but each strategy adds synthetic steps and cost [225, 226]. The route of administration is the more fundamental obstacle. Oral bioavailability remains low because of gastrointestinal proteolysis and limited epithelial permeability, and although permeation enhancers, enzyme inhibitors, and targeted carriers have improved absorption, no general solution has emerged [227, 228]. Because treatment in endemic settings must be compatible with practical delivery and limited infrastructure, a parenteral‐only candidate can face a disadvantage independent of potency [229, 230].

### 7.3 Immunogenicity and Carrier‐Related Immune Responses

Peptides are frequently described as poorly immunogenic, but this generalization does not survive scrutiny at the level of the drug product. Synthesis‐related impurities can introduce novel T‐cell epitopes, and regulatory frameworks now require formal immunogenicity risk assessment of impurity profiles rather than of the target sequence alone [231]. When peptides are delivered on nanocarriers, the immunological liability shifts to the carrier. Preexisting and treatment‐induced anti‐PEG antibodies alter biodistribution, accelerate blood clearance, and cause hypersensitivity in a subset of individuals, and mechanistic work shows that anti‐PEG antibodies activate complement, compromise bilayer integrity, and cause premature cargo release from PEGylated liposomes and lipid nanoparticles [232, 233]. A related and less tractable issue is the biomolecular corona: The protein layer acquired in vivo is determined jointly by particle surface chemistry and by the biological milieu, and it can mask targeting ligands, redirecting particles away from their intended cells [234, 235]. For peptide‐decorated particles, this is a direct threat to the targeting rationale, and it is aggravated in endemic populations, where hypergammaglobulinemia, chronic parasitemia, and frequent co‐infection produce plasma proteomes that differ from those of the healthy volunteers in whom carriers are typically characterized. This creates a clear need to characterize corona formation directly in patient‐derived plasma from severe malaria and visceral leishmaniasis rather than assuming that data from healthy plasma will transfer [234, 235].

### 7.4 Manufacturing, Cost of Goods, and Environmental Footprint

Manufacturing economics determine whether a candidate is deployable in the settings where these diseases occur. Solid‐phase peptide synthesis remains the dominant platform but consumes large volumes of solvent and reagent; a cross‐industry analysis of process mass intensity across 14 companies quantified this footprint and identified it as the principal sustainability constraint of the modality [236]. Process innovation is addressing this limitation. Complete elimination of the washing steps in each coupling cycle has been demonstrated at the research and production scale for sequences up to 89 residues, reducing waste by up to 95% while requiring only 10%–15% of the standard amount of base, and liquid‐phase synthesis on soluble supports offers a complementary route to large‐scale production with reduced solvent consumption [237, 238]. Comparable progress exists on the nanocarrier side, where microfluidic platforms now span flow rates from exploratory to industrial scale without loss of mixing control, and automated parallelized systems generate on the order of 1,000 distinct lipid nanoparticle formulations per hour [239, 240]. These advances do not remove the core difficulty of combination products. Analysis of nanoparticle‐based agents that entered clinical trials identifies the complexity of multifunctional constructs as a primary translational barrier, because it introduces batch‐to‐batch variability and unpredictable in vivo behavior [241]. A peptide‐decorated nanocarrier inherits the control burden of both components, and each additional functional element must therefore be justified by a demonstrated gain rather than by design elegance.

### 7.5 Regulatory and Quality Requirements

The regulatory expectations for synthetic peptides are more demanding than the preclinical literature generally acknowledges. Enantiomeric purity must be demonstrated because racemization during synthesis generates D‐isomer impurities that alter activity and safety, and well‐characterized reference standards, established through multi‐laboratory value assignment using NMR, mass spectrometry, and chromatography, are required to support identity, purity, and strength determinations [242, 243]. For generic products, abbreviated approval pathways place the analytical burden squarely on impurity characterization and comparability [215]. None of these requirements is prohibitive, but they arrive late in development and are rarely anticipated in academic candidate selection, which typically optimizes potency and selectivity alone [244]. Recent syntheses of the field reach the same conclusion from an industrial perspective. Enzymatic degradation, limited permeability, and reliance on parenteral administration remain the dominant constraints, and addressing them at the design stage is more effective than retrofitting solutions after a lead has been nominated [245].

### 7.6 Cost‐Effectiveness and Deployability in Endemic Settings

Even a technically successful product must satisfy an economic constraint that is unusually strict in these indications. Target product profiles for severe malaria specify rapid parasiticidal onset, a low propensity for resistance selection, and compatibility with existing combination partners, alongside affordability and suitability for use in district‐level facilities, and the corresponding profile for cutaneous leishmaniasis diagnostics sets explicit ceilings on cost and infrastructure [25, 229]. Economic modeling of malaria‐adjacent screening shows that the cost‐effectiveness of a diagnostic strategy depends strongly on local prevalence, so that a platform that is cost‐effective in a high‐transmission district may not be so in a low‐prevalence one [246]. For biosensors, the translation literature identifies manufacturability, shelf stability, and integration into existing clinical pathways—rather than analytical performance—as the determinants of adoption [247]. Applied to peptide‐enabled nanoplatforms, these constraints imply that a candidate whose cost of goods exceeds that of a conventional rapid diagnostic test or a generic antiparasitic drug will not be adopted, regardless of its analytical or pharmacological superiority. It is notable that the most advanced recent preclinical candidate for visceral leishmaniasis is an orally bioavailable small molecule selected explicitly against such a profile; peptide‐based programs will be judged against that benchmark [230].

### 7.7 Improving Peptide Developability

Several developments could lower attrition if adopted early. Machine learning now supports sequence identification and optimization with experimentally validated output [248]. A sequence‐optimized peptide developed by this route showed efficacy against drug‐resistant *S. aureus* in mice, while agent‐based generative approaches have produced D‐enantiomeric peptides that combine protease resistance with in vivo activity [249, 250]. Structure‐aware deep learning models allow toxicity to be predicted before synthesis, shifting the selectivity filter upstream of the wet laboratory campaign [251]. Combination design offers a further safeguard. Random peptide mixtures and defined combinations suppress the evolution of resistance more effectively than single sequences, which is relevant given the resistance selection observed with murepavadin [252]. On the delivery side, surveys of nanocarrier‐enabled AMPs indicate that encapsulation and molecular modification are complementary rather than alternative routes, and reviews of clinical translation status converge on the same set of blocking factors identified here [253, 254]. The geometry of peptide presentation also deserves more attention than it currently receives. Ligand valency has been shown to determine tissue accumulation of peptide‐targeted nanoparticles, while spacer length and composition govern targeting specificity in peptide–nanoparticle conjugates [255, 256]. These are experimentally tractable parameters that should be optimized explicitly in antiparasitic studies.

These barriers help explain the gap between the large preclinical literature and the small number of clinically advanced candidates. The evidence assembled here supports a practical hierarchy for candidate selection in malaria and leishmaniasis: measured stability in serum and in the relevant intracellular compartment first, then a realistic administration route, then carrier immunogenicity and corona behavior in patient‐derived matrices, and only then optimization of potency. Candidates that cannot satisfy the first three criteria are unlikely to reach the clinic, irrespective of their in vitro performance.

## 8 Conclusions and Future Perspectives

Malaria and leishmaniasis remain important causes of morbidity and mortality in low‐ and middle‐income countries, with current chemotherapeutic options constrained by long treatment schedules, cumulative toxicity, and documented resistance to antimonials, amphotericin B formulations, miltefosine, and artemisinin derivatives. The work summarized here shows that peptide‐based approaches, when combined with nanocarriers, provide practical means to adjust where and how antiparasitic agents act. Cell‐penetrating and membrane‐active peptides, peptoids, and mitochondria‐directed motifs have been used both as active compounds and as vectors for small molecules or biologics, improving access to hepatocytes, erythrocytes, macrophages, and parasitophorous vacuoles. Incorporating these sequences into lipidic, polymeric, metallic, or silica‐based nanoparticles adds control over intracellular delivery and exposure time, which helps to separate antiparasitic effects from host toxicity in preclinical models. A realistic assessment must nevertheless acknowledge that none of the peptide‐based strategies reviewed here has entered clinical evaluation for malaria or leishmaniasis, and that the barriers analyzed in Section 7—proteolytic instability, unfavorable pharmacokinetics, carrier immunogenicity, manufacturing cost, and regulatory burden—are the principal reason. Progress will depend less on the discovery of additional active sequences than on the systematic generation of the data types that currently decide attrition: stability and pharmacokinetics measured in physiologically relevant matrices, corona and immunogenicity characterization in patient‐derived plasma, and cost modeling against published target product profiles from the outset of candidate selection.

Nanocarriers decorated with mannose, tuftsin, tachyplesin analogues, or defined immunoreactive fragments tend to accumulate in organs rich in mononuclear phagocytes and in skin or mucosal lesions while protecting labile peptide sequences and allowing sustained or triggered release. The main uncertainties that now limit translation concern the behavior in complex tissues rather than formulation per se. Three areas emerge as priorities for experimental work: establishing quantitative relationships between in vitro release profiles and pharmacodynamic responses in granulomatous or fibrotic environments; defining how protein coronas evolve and how peptide‐modified nanoparticles traffic through endolysosomal compartments during infection; and determining how repeated or suboptimal dosing affects local immunity and the selection of tolerant or resistant parasite populations. Addressing these points will require combined use of advanced imaging, realistic infection models, and pharmacokinetic/pharmacodynamic analysis. Diagnostic developments follow a related logic. Aptamers and synthetic peptides integrated into electrochemical, fluorescent, or plasmonic nanosensors based on gold nanoparticles, quantum dots, metal–organic frameworks, or graphene derivatives already reach detection limits compatible with low‐parasitemia malaria and with specific identification of leishmaniasis in co‐endemic settings. Peptide antigens and aptamers directed to LDH, GDH, GP63, KMP‐11, and kDNA offer defined composition and thermal stability that are useful for deployment outside reference laboratories. The critical next step is systematic validation rather than further incremental gains in analytical sensitivity. Application of STARD and REASSURED criteria, testing in clinically relevant matrices such as capillary blood, lesion swabs, serum, and urine, and long‐term stability assessment under temperature and humidity conditions typical of endemic regions are needed before these platforms can be incorporated into surveillance programs. Further progress in antiparasitic nanomedicine will depend on closer interaction between peptide chemistry, nanomaterial design, and systems‐level analysis. Omics data, structural models, and machine‐learning selection of peptide candidates can be integrated with pharmacokinetic and pharmacodynamic studies to define organelles, receptors, and immune pathways that are suitable targets for both therapy and diagnosis. Modular synthetic routes and orthogonal conjugation strategies can turn peptide motifs into interchangeable units on shared nanoparticle backbones, facilitating adjustment to parasite species, clinical form, and regional resistance patterns without complete reformulation. Controlled human infection studies and well‐characterized longitudinal cohorts in endemic areas provide the setting in which efficacy, safety, relapse, and resistance can be measured under field‐relevant conditions. Under these circumstances, peptides may serve as useful building blocks for future theranostic systems that link prevention, case detection, and targeted treatment of parasitic diseases in resource‐constrained health services.

## Author Contributions

**Christian S. Carnero Canales:** writing – review and editing, writing – original draft, validation, investigation, conceptualization, supervision, resources, project administration, funding acquisition. **Ana Clara Lunardi Yagi** and **Túlio Custódio Reis:** writing – original draft, visualization, validation, investigation, and formal analysis. **Jessica Ingrid Cazorla** and **Jade Rojas Villar:** writing – original draft, investigation, formal analysis. **Marcia A. Graminha** writing – review and editing, validation, supervision, conceptualization. **Roxana Yesenia Pastrana Alta** and **Fernando Rogério Pavan:** writing – review and editing, visualization, validation, supervision, resources, project administration, funding acquisition, conceptualization.

## Funding

This study was supported by the National Program for Scientific Research and Advanced Studies (E077‐2023‐01‐BM, PE501092284‐2024, E033‐2023‐01‐BM), Interinstitutional Alliances for Doctoral Programs (PE501084300‐2023‐PROCIENCIA‐BM), Fundação de Amparo à Pesquisa do Estado de São Paulo (2023/01664‐1, 2025/17976‐8), and Coordenação de Aperfeiçoamento de Pessoal de Nível Superior (001).

## Conflicts of Interest

The authors declare no conflicts of interest.

## Data Availability

No new data were generated or analyzed in this review. All data discussed are available in the cited literature.

## References

[1] Q. Li , T. Liu , K. Lv , et al., “Malaria: Past, Present, and Future,” Signal Transduction and Targeted Therapy 10 (2025): 188, 10.1038/s41392-025-02246-3. 40523953 PMC12170910

[2] M. D. Conrad , V. Asua , S. Garg , et al., “Evolution of Partial Resistance to Artemisinins in Malaria Parasites in Uganda,” New England Journal of Medicine 389 (2023): 722–732, 10.1056/NEJMoa2211803. 37611122 PMC10513755

[3] S. Mihreteab , K. Anderson , I. M. de la Fuente , et al., “The Spread of Molecular Markers of Artemisinin Partial Resistance and Diagnostic Evasion in Eritrea: A Retrospective Molecular Epidemiology Study,” Lancet Microbe 6 (2025): 100930, 10.1016/S2666-5247(24)00172-1. 39653047 PMC11798904

[4] W. Sheahan , A. Golden , R. Barney , et al., “Estimating Malaria Antigen Dynamics and the Time to Negativity of Next‐Generation Malaria Rapid Diagnostic Tests,” Malaria Journal 24 (2025): 109, 10.1186/s12936-025-05350-5. 40186233 PMC11969773

[5] H. Ntuku , B. Whittemore , L. Dausab , et al., “Post‐Treatment Duration of Positivity for Standard and Ultra‐Sensitive Plasmodium Falciparum Antigen‐Based Rapid Diagnostic Tests, a Cohort Study From a Low‐Endemic Setting in Namibia,” EBioMedicine 111 (2025): 105489, 10.1016/j.ebiom.2024.105489. 39657364 PMC11683224

[6] H. J. C. de Vries and H. D. Schallig , “Cutaneous Leishmaniasis: A 2022 Updated Narrative Review Into Diagnosis and Management Developments,” American Journal of Clinical Dermatology 23 (2022): 823–840, 10.1007/s40257-022-00726-8. 36103050 PMC9472198

[7] F. Frézard , M. M. G. Aguiar , L. A. M. Ferreira , et al., “Liposomal Amphotericin B for Treatment of Leishmaniasis: From the Identification of Critical Physicochemical Attributes to the Design of Effective Topical and Oral Formulations,” Pharmaceutics 15 (2022): 99, 10.3390/pharmaceutics15010099. 36678729 PMC9864876

[8] A. K. Gupta , S. Das , M. Kamran , S. A. Ejazi , and N. Ali , “The Pathogenicity and Virulence of Leishmania ‐ Interplay of Virulence Factors With Host Defenses,” Virulence 13 (2022): 903–935, 10.1080/21505594.2022.2074130. 35531875 PMC9154802

[9] J. P. Assolini , A. C. M. Carloto , B. T. da S. Bortoleti , et al., “Nanomedicine in Leishmaniasis: A Promising Tool for Diagnosis, Treatment and Prevention of Disease ‐ An Update Overview,” European Journal of Pharmacology 923 (2022): 174934, 10.1016/j.ejphar.2022.174934. 35367420

[10] J. X. Zheng , Y. Liu , S. Y. Guan , et al., “Global, Regional, and National Burden of Neglected Tropical Diseases and Malaria in the General Population, 1990‐2021: Systematic Analysis of the Global Burden of Disease Study 2021,” Journal of Advanced Research 79 (2026): 769–781, 10.1016/j.jare.2025.04.004. 40194698 PMC12766246

[11] Y. S. Zhu , Z. S. Sun , J. X. Zheng , et al., “Prevalence and Attributable Health Burdens of Vector‐Borne Parasitic Infectious Diseases of Poverty, 1990–2021: Findings From the Global Burden of Disease Study 2021,” Infectious Diseases of Poverty 13 (2024): 96, 10.1186/s40249-024-01260-x. 39658783 PMC11633012

[12] J. Li , H. J. Docile , D. Fisher , K. Pronyuk , and L. Zhao , “Current Status of Malaria Control and Elimination in Africa: Epidemiology, Diagnosis, Treatment, Progress and Challenges,” Journal of epidemiology and global health 14 (2024): 561–579, 10.1007/s44197-024-00228-2. 38656731 PMC11442732

[13] P. Venkatesan , “WHO World Malaria Report 2024,” Lancet Microbe 2025, 101073. 39923782 10.1016/j.lanmic.2025.101073

[14] S. X. Zhang , G. B. Yang , J. Y. Sun , et al., “Global, Regional, and National Burden of Visceral Leishmaniasis, 1990–2021: Findings From the Global Burden of Disease Study 2021,” Parasites & Vectors 18 (2025): 157, 10.1186/s13071-025-06796-x. 40287729 PMC12032768

[15] E. S. D. Reis , W. S. Paz , R. E. S. Ramos , et al., “Spatial and Temporal Modeling of the Global Burden of Cutaneous Leishmaniasis in Brazil: A 21‐Year Ecological Study,” PLoS Neglected Tropical Diseases 18 (2024): e0012668. 39565743 10.1371/journal.pntd.0012668 PMC11578532

[16] M. Pareyn , F. Alves , S. Burza , et al., “Leishmaniasis,” Nature Reviews Disease Primers 11 (2025): 81. 10.1038/s41572-025-00663-w 41266459

[17] T. Sargsyan , L. Stepanyan , A. Tsaturyan , R. Palumbo , C. Vicidomini , and G. N. Roviello , “Intracellular Parasitic Infections Caused by Plasmodium Falciparum, Leishmania Spp., Toxoplasma Gondii, Echinococcus Multilocularis, Among Key Pathogens: Global Burden, Transmission Dynamics, and Vaccine Advances—a Narrative Review,” Vaccines 13 (2025): 1082, 10.3390/vaccines13111082. 41295456 PMC12656568

[18] M. Maurizio , M. Masid , K. Woods , et al., “Host Cell CRISPR Genomics and Modelling Reveal Shared Metabolic Vulnerabilities in the Intracellular Development of Plasmodium Falciparum and Related Hemoparasites,” Nature Communications 15 (2024): 6145, 10.1038/s41467-024-50405-x. PMC11271486 39034325

[19] S. Musumeci , A. Kruse , F. Chappuis , T. O. Jensen , and G. Alcoba , “Neglected Etiologies of Prolonged Febrile Illnesses in Tropical and Subtropical Regions: A Systematic Review,” PLoS Neglected Tropical Diseases 18 (2024): e0011978, 10.1371/journal.pntd.0011978. 38905305 PMC11221637

[20] U. Ornellas‐Garcia , L. Freire‐Antunes , M. Rangel‐Ferreira , et al., “Impact of Co‐Infection With Plasmodium Berghei ANKA in Leishmania Major‐Parasitized Mice on Immune Modulation and Cutaneous Leishmaniasis,” PLoS Neglected Tropical Diseases 19 (2025): e0013302, 10.1371/journal.pntd.0013302. 40720541 PMC12316397

[21] E. Ortiz‐Prado , J. Vasconez‐Gonzalez , J. C. Pazmiño‐Almeida , et al., “Climate Change and the Rising Threat of Vector‐Borne Diseases in the Andes,” One Health 22 (2026): 101362, 10.1016/j.onehlt.2026.101362. 41737197 PMC12926586

[22] P. J. Rosenthal , V. Asua , J. A. Bailey , et al., “The Emergence of Artemisinin Partial Resistance in Africa: How Do We Respond?,” The Lancet Infectious Diseases 24 (2024): e591–e600, 10.1016/S1473-3099(24)00141-5. 38552654 PMC12954456

[23] G. D. Ghousepeer , M. Rani , A. Kumar , et al., “Insights Into Drug Resistance in Leishmania: Mechanisms, Therapeutics, and Clinical Case Studies,” ADMET & DMPK 14 (2025): 2992, 10.5599/admet.2992. PMC12994601 41853234

[24] J. M. Louis , E. Mazigo , H. Jun , et al., “First Report of pfhrp2 and pfhrp3 Gene Deletions Compromising HRP2‐Based Malaria Rapid Diagnostic Tests in Malawi,” Infectious Diseases of Poverty 14 (2025): 98, 10.1186/s40249-025-01368-8. 41068992 PMC12512526

[25] J. Jarzabek and P. W. Denny , “Molecular Diagnostics for Cutaneous Leishmaniasis: Progress Towards Fulfilling the WHO Target Product Profile,” Parasitology 153 (2025): 1–19, 10.1017/S0031182025101467. PMC13215747 41449489

[26] D. Ochol , K. Haile , N. Onduma , T. Yohanes , and K. Kamara , “Elimination of Visceral Leishmaniasis in Ethiopia: Cross‐Sector Collaboration and Cost Sharing to Promote Sustainability,” International Journal of Infectious Diseases 152 (2025): 107800, 10.1016/j.ijid.2025.107800. 39864498

[27] S. Sundar , “The Story of Elimination of Visceral Leishmaniasis (Kala‐Azar) in India—Challenges Towards Sustainment,” PLoS Neglected Tropical Diseases 19 (2025): e0013321, 10.1371/journal.pntd.0013321. 40828749 PMC12364345

[28] L. L. G. Ferreira , J. de Moraes , and A. D. Andricopulo , “Approaches to Advance Drug Discovery for Neglected Tropical Diseases,” Drug Discovery Today 27 (2022): 2278–2287, 10.1016/j.drudis.2022.04.004. 35398562

[29] A. Cesaro , S. Lin , N. Pardi , and C. de la Fuente‐Nunez , “Advanced Delivery Systems for Peptide Antibiotics,” Advanced Drug Delivery Reviews 196 (2023): 114733, 10.1016/j.addr.2023.114733. 36804008 PMC10771258

[30] J. Liu , H. Cabral , and P. Mi , “Nanocarriers Address Intracellular Barriers for Efficient Drug Delivery, Overcoming Drug Resistance, Subcellular Targeting and Controlled Release,” Advanced Drug Delivery Reviews 207 (2024): 115239, 10.1016/j.addr.2024.115239. 38437916

[31] B. Beitzinger , B. Draphoen , J. Hald , et al., “On the Cellular Selectivity of Targeting Peptide‐Nanoparticle Conjugates – a Nanoparticle Perspective,” Expert Opinion on Drug Delivery 22 (2025): 1345–1358, 10.1080/17425247.2025.2522245. 40528422

[32] Z. He , C. Yu , Z. Pan , et al., “Erythrocyte Membrane With CLIPPKF as Biomimetic Nanodecoy Traps Merozoites and Attaches to Infected Red Blood Cells to Prevent Plasmodium Infection,” Journal of Nanobiotechnology 21 (2023): 15, 10.1186/s12951-022-01709-x. 36647056 PMC9841648

[33] V. T. Hoang , H. Hong , T.‐H. Eom , H. Park , and S.‐J. Yeo , “A Novel Peptide Pair‐Based Rapid Fluorescent Diagnostic System for Malaria Plasmodium Falciparum Detection,” Talanta 281 (2025): 126828, 10.1016/j.talanta.2024.126828. 39265425

[34] B. A. Braz , M. Hospinal‐Santiani , G. Martins , et al., “Disposable Electrochemical Platform Based on Solid‐Binding Peptides and Carbon Nanomaterials: An Alternative Device for Leishmaniasis Detection,” Microchimica Acta 190 (2023): 321, 10.1007/s00604-023-05891-z. 37491620

[35] A. S. Gozalo , C. K. Robinson , J. Holdridge , O. L. Franco Mahecha , and W. R. Elkins , American Association for Laboratory Animal Science preprint, 2024, 10.30802/AALAS-CM-24-000019

[36] I. S. Walker and S. J. Rogerson ,“Pathogenicity and Virulence of Malaria: Sticky Problems and Tricky Solutions,” Virulence 14 (2023): 2150456, 10.1080/21505594.2022.2150456. 36419237 PMC9815252

[37] J. D. Dvorin and D. E. Goldberg , “Plasmodium Egress Across the Parasite Life Cycle,” Annual Review of Microbiology 76 (2022): 67–90, 10.1146/annurev-micro-041320-020659. 35417197

[38] E. Meibalan and M. Marti , “Biology of Malaria Transmission,” Cold Spring Harbor Perspectives in Medicine 7 (2017): a025452, 10.1101/cshperspect.a025452. 27836912 PMC5334247

[39] P. J. Rosenthal , V. Asua , and M. D. Conrad , “Emergence, Transmission Dynamics and Mechanisms of Artemisinin Partial Resistance in Malaria Parasites in Africa,” Nature Reviews. Microbiology 22 (2024): 373–384, 10.1038/s41579-024-01008-2. 38321292

[40] A. G. Maier , K. Matuschewski , M. Zhang , and M. Rug , “Plasmodium Falciparum,” Trends in Parasitology 35 (2019): 481–482, 10.1016/j.pt.2018.11.010. 30595467

[41] About Malaria, Malaria, CDC, n.d, (accessed 28 November 2025), https://www.cdc.gov/malaria/about/index.html.

[42] G. Pradel , S. Garapaty , and U. Frevert , “Proteoglycans Mediate Malaria Sporozoite Targeting to the Liver,” Molecular Microbiology 45 (2002): 637–651, 10.1046/j.1365-2958.2002.03057.x. 12139612

[43] M. Jacobs‐Lorena and S. J. Cha , “Unbiased Phage Display Screening Identifies Hidden Malaria Vaccine Targets,” Emerging Microbes & Infections 13 (2024): 2429617, 10.1080/22221751.2024.2429617. 39529575 PMC11587725

[44] S. J. Cha , M. S. Kim , C. H. Na , and M. Jacobs‐Lorena , “Plasmodium Sporozoite Phospholipid Scramblase Interacts With Mammalian Carbamoyl‐Phosphate synthetase 1 to Infect Hepatocytes,” Nature Communications 12 (2021): 6773, 10.1038/s41467-021-27109-7. PMC8604956 34799567

[45] S. J. Cha , K. Park , P. Srinivasan , et al., “CD68 Acts as a Major Gateway for Malaria Sporozoite Liver Infection,” Journal of Experimental Medicine 212 (2015): 1391–1403, 10.1084/jem.20110575. 26216124 PMC4548058

[46] A. S. P. Yang , S. Lopaticki , M. T. O’Neill , et al., “AMA1 and MAEBL Are Important for Plasmodium Falciparum Sporozoite Infection of the Liver,” Cellular Microbiology 19 (2017): e12745, 10.1111/cmi.12745. 28371168

[47] L. Aguiar , A. Biosca , E. Lantero , et al., “Coupling the Antimalarial Cell Penetrating Peptide TP10 to Classical Antimalarial Drugs Primaquine and Chloroquine Produces Strongly Hemolytic Conjugates,” Molecules 24 (2019): 4559, 10.3390/molecules24244559. 31842498 PMC6943437

[48] J. Molina‐Franky , M. E. Patarroyo , M. Kalkum , and M. A. Patarroyo , “The Cellular and Molecular Interaction Between Erythrocytes and Plasmodium Falciparum Merozoites,” Frontiers in Cellular and Infection Microbiology 12 (2022): 816574, 10.3389/fcimb.2022.816574. 35433504 PMC9008539

[49] T. C. Reis , A. C. L. Yagi , A. L. D. Ramos , A. M. A. Velásquez , N. C. C. Coelho , and M. A. S. Graminha , “Surface Molecules of Leishmania: From Virulence Determinants to Therapeutic and Vaccine Targets,” Molecular and Biochemical Parasitology (2025): 111702, 10.1016/j.molbiopara.2025.111702. 41033399

[50] T. C. Reis , A. C. L. Yagi , A. M. A. Velásquez , et al., “Functional Effects of Cyclization on Li1 Peptide Activity Against Metacyclic, *Leishmania Amazonensis*, Internalization,” ACS Omega 11 (2026): 16164–16176, 10.1021/acsomega.5c11292. 41867605 PMC13000623

[51] I. R. Palombi , N. Lawrence , A. M. White , et al., “Development of Antiplasmodial Peptide‐Drug Conjugates Using a Human Protein‐Derived Cell‐Penetrating Peptide With Selectivity for Infected Cells,” Bioconjugate Chemistry 34 (2023): 1105–1113, 10.1021/acs.bioconjchem.3c00147. 37232456

[52] A. F. Silva , M. D. T. Torres , L. S. Silva , et al., “Synthetic Angiotensin II Peptide Derivatives Confer Protection Against Cerebral and Severe Non‐Cerebral Malaria in Murine Models,” Scientific Reports 14 (2024): 4682, 10.1038/s41598-024-51267-5. 38409185 PMC10897374

[53] S. Somsri , M. Mungthin , N. Klubthawee , P. Adisakwattana , W. Hanpithakpong , and R. Aunpad , “A Mitochondria‐Penetrating Peptide Exerts Potent Anti‐Plasmodium Activity and Localizes at Parasites’ Mitochondria,” Antibiotics 10 (2021): 1560, 10.3390/antibiotics10121560. 34943772 PMC8698686

[54] K. Singh , R. Ranjha , W. A. Malla , et al., “Ligand Decorated Nanostructure Carrier Systems for Targeted Delivery of Antimalarials,” Discover Nano 21 (2026): 6, 10.1186/s11671-026-04695-3. 42225854 PMC13226761

[55] Leishmaniasis, n.d., (Accessed 28 November 2025), https://www.who.int/news‐room/fact‐sheets/detail/leishmaniasis.

[56] Leishmaniasis ‐ PAHO/WHO | Pan American Health Organization, n.d., (Accessed 28 November 2025) https://www.paho.org/en/topics/leishmaniasis.

[57] R. Arenas , E. Torres‐Guerrero , M. R. Quintanilla‐Cedillo , and J. Ruiz‐Esmenjaud , “Leishmaniasis: A Review,” F1000Research 6 (2017): 750, 10.12688/f1000research. 28649370 PMC5464238

[58] B. M. Roatt , J. M. de Oliveira Cardoso , R. C. F. De Brito , W. Coura‐Vital , R. D. de Oliveira Aguiar‐Soares , and A. B. Reis , “Recent Advances and New Strategies on Leishmaniasis Treatment,” Applied Microbiology and Biotechnology 104 (2020): 8965–8977, 10.1007/s00253-020-10856-w. 32875362

[59] S. Sundar , J. Singh , V. K. Singh , N. Agrawal , and R. Kumar , “Current and Emerging Therapies for the Treatment of Leishmaniasis,” Expert Opinion on Orphan Drugs 12 (2024): 19–32, 10.1080/21678707.2024.2335248.

[60] S. Tambe , S. Nag , S. R. Pandya , et al., “Revolutionizing Leishmaniasis Treatment With Cutting Edge Drug Delivery Systems and Nanovaccines: An Updated Review,” ACS Infectious Diseases 10 (2024): 1871–1889, 10.1021/acsinfecdis.4c00010. 38829047

[61] J. Young and P. E. Kima , “The Leishmania Parasitophorous Vacuole Membrane at the Parasite‐Host Interface,” Yale Journal of Biology and Medicine 92 (2019): 511, 10.1371/journal.pone.0035671. 31543712 PMC6747952

[62] A. P. Nunes , Y. M. dos Santos‐Destro , A. C. J. Rodrigues , et al., “Under Pressure: Updated Insights Into the Mechanisms of Leishmania's Defense in Response to Oxidative Stress,” Life Sciences 377 (2025): 123779, 10.1016/j.lfs.2025.123779. 40460927

[63] V. S. Cavalcante‐Costa , M. Costa‐Reginaldo , T. Queiroz‐Oliveira , et al., “Leishmania Amazonensis Hijacks Host Cell Lysosomes Involved in Plasma Membrane Repair to Induce Invasion in Fibroblasts,” Journal of Cell Science 132 (2019), 10.1242/JCS.226183/VIDEO-2. 30814331

[64] A. Schwing , D. F. Pisani , C. Pomares , et al., “Identification of Adipocytes as Target Cells for Leishmania Infantum Parasites,” Scientific Reports 11 (2021): 21275, 10.1038/s41598-021-00443-y. 34711872 PMC8553825

[65] F. Tomiotto‐Pellissier , B. T. da S. Bortoleti , J. P. Assolini , et al., “Macrophage Polarization in Leishmaniasis: Broadening Horizons,” Frontiers in Immunology 9 (2018): 2529, 10.3389/fimmu.2018.02529. 30429856 PMC6220043

[66] A. Saha , S. Roy , and A. Ukil , “Cytokines and Signaling Networks Regulating Disease Outcomes in Leishmaniasis,” Infection and Immunity 90 (2022): 1–12, 10.1128/IAI.00248-22. PMC9387244 35862725

[67] F. S. Almeida , S. E. R. Vanderley , F. C. Comberlang , et al., “Leishmaniasis: Immune Cells Crosstalk in Macrophage Polarization,” Tropical Medicine and Infectious Disease 8 (2023): 276, 10.3390/tropicalmed8050276. 37235324 PMC10222886

[68] V. Liévin‐Le Moal , and P. M. Loiseau , “ *Leishmania* Hijacking of the Macrophage Intracellular Compartments,” FEBS Journal 283 (2016): 598–607, 10.1111/febs.2016.283.issue-4. 26588037

[69] H. Goto and J. A. L. Lindoso , “Current Diagnosis and Treatment of Cutaneous and Mucocutaneous Leishmaniasis,” Expert Review of Anti‐Infective Therapy 8 (2014): 419–433, 10.1586/eri.10.19. 20377337

[70] B. M. Scorza , E. M. Carvalho , and M. E. Wilson , “Cutaneous Manifestations of Human and Murine Leishmaniasis,” International Journal of Molecular Sciences 18 (2017): 1296. 28629171 10.3390/ijms18061296 PMC5486117

[71] M. S. Gurel , B. Tekin , and S. Uzun , “Cutaneous Leishmaniasis: A Great Imitator,” Clinics in Dermatology 38 (2020): 140–151, 10.1016/j.clindermatol.2019.10.008. 32513395

[72] P. Kaye and P. Scott , “Leishmaniasis: Complexity at the Host–pathogen Interface,” Nature Reviews Microbiology 9 (2011): 604–615, 10.1038/nrmicro2608. 21747391

[73] B. Alemayehu and M. Alemayehu , “Leishmaniasis: A Review on Parasite, Vector and Reservoir Host,” Health Science Journal 11 (2017): 519, 10.21767/1791-809x.1000519.

[74] S. Mann , K. Frasca , S. Scherrer , et al., “A Review of Leishmaniasis: Current Knowledge and Future Directions,” Current Tropical Medicine Reports 8 (2021): 121, 10.1007/s40475-021-00232-7. 33747716 PMC7966913

[75] V. B. G. Porto , L. B. Carvalho , B. F. Buzo , et al., “Visceral Leishmaniasis Caused by Leishmania (Leishmania) Amazonensis Associated With Hodgkin's Lymphoma,” Revista Do Instituto De Medicina Tropical De Sao Paulo 64 (2022): e51–e51, 10.1590/S1678-9946202264051. 36074446 PMC9448255

[76] A. Özbilgin , V. Tunalı , İ. Çavuş , et al., “Visceral Leishmaniasis Caused by Leishmania Tropica,” Acta Parasitologica 68 (2023): 699–704, 10.1007/s11686-023-00695-w. 37351773

[77] S. Scarpini , A. Dondi , C. Totaro , et al., “Visceral Leishmaniasis: Epidemiology, Diagnosis, and Treatment Regimens in Different Geographical Areas With a Focus on Pediatrics,” Microorganisms 10 (2022): 1887, 10.3390/microorganisms10101887. 36296164 PMC9609364

[78] P. Kumar , M. Chatterjee , and N. Das , “Post Kala‐Azar Dermal Leishmaniasis: Clinical Features and Differential Diagnosis,” Indian Journal of Dermatology 66 (2021): 24, 10.4103/ijd.IJD_602_20. 33911290 PMC8061484

[79] I. S. Lima , M. S. Solcá , W. L. Tafuri , W. L. C. Dos‐Santos , and L. A. R. De Freitas , “Assessment of Histological Liver Alterations in Dogs Naturally Infected With Leishmania Infantum,” Parasites & Vectors 12 (2019), 10.1186/s13071-019-3723-1. PMC6796381 31619264

[80] A. Poulaki , E. T. Piperaki , and M. Voulgarelis , “Effects of Visceralising Leishmania on the Spleen, Liver, and Bone Marrow: A Pathophysiological Perspective,” Microorganisms 9 (2021): 759, 10.3390/microorganisms9040759. 33916346 PMC8066032

[81] V. Kumar and A. Chugh , “Peptide‐Mediated Leishmaniasis Management Strategy: Tachyplesin Emerges as an Effective Anti‐Leishmanial Peptide Against Leishmania Donovani,” Biochimica et Biophysica Acta (BBA) ‐ Biomembranes 1863 (2021): 183629, 10.1016/j.bbamem.2021.183629. 33933430

[82] V. Kumar , J. S. Lin , N. Molchanova , et al., “Membrane‐Acting Biomimetic Peptoids Against Visceral Leishmaniasis,” FEBS Open Bio 13 (2023): 519–531, 10.1002/feb4.v13.3. PMC9989931 36683396

[83] C. K. Bhusal , S. Sinha , D. Kaur , and R. Sehgal , “Unravelling Drug Resistance in Leishmaniasis: Genomic Adaptations and Emerging Therapies,” Frontiers in Molecular Biosciences 12 (2025), 10.3389/fmolb.2025.1573618. PMC12146366 40492115

[84] A. Ponte‐Sucre , F. Gamarro , J.‐C. Dujardin , et al., “Drug Resistance and Treatment Failure in Leishmaniasis: A 21st Century Challenge,” PLoS Neglected Tropical Diseases 11 (2017): e0006052, 10.1371/journal.pntd.0006052. 29240765 PMC5730103

[85] A. M. Arenas Velásquez , I. A. Patino Linares , L. D. Gaspers , et al., “The Binuclear Cyclopalladated Complex CP2 Is Targeting Ubiquinol‐Cytochrome c Reductase (complex III) of Leishmania Amazonensis,” International Journal for Parasitology: Drugs and Drug Resistance 27 (2025): 100574, 10.1016/j.ijpddr.2024.100574. 39746288 PMC11748178

[86] G.‐J. Wijnant , F. Dumetz , L. Dirkx , et al., “Tackling Drug Resistance and Other Causes of Treatment Failure in Leishmaniasis,” Frontiers in Tropical Diseases 3 (2022), 837460, 10.3389/fitd.2022.837460.

[87] F. J. Pérez‐Victoria , M. P. Sánchez‐Cañete , S. Castanys , and F. Gamarro , “Phospholipid Translocation and Miltefosine Potency Require Both L. Donovani Miltefosine Transporter and the New Protein LdRos3 in Leishmania Parasites,” Journal of Biological Chemistry 281 (2006): 23766–23775, 10.1074/jbc.M605214200. 16785229

[88] F. J. Pérez‐Victoria , S. Castanys , and F. Gamarro , “ *Leishmania Donovani* Resistance to Miltefosine Involves a Defective Inward Translocation of the Drug,” Antimicrobial Agents and Chemotherapy 47 (2003): 2397–2403, 10.1128/AAC.47.8.2397-2403.2003. 12878496 PMC166066

[89] M. Fekrisoofiabadi , M. Fekri , A. Moradabadi , et al., “Evaluation of MDR1 and MRPA Genes Expression in Different Types of Dry Cutaneous Leishmaniasis,” BMC Research Notes 12 (2019): 803, 10.1186/s13104-019-4784-0. 31831065 PMC6909633

[90] M. B. R. de Santana , G. O. Miranda , and L. P. Carvalho , “ATP‐Binding Cassette Transporters and Drug Resistance in Cutaneous Leishmaniasis,” International Journal of Infectious Diseases 151 (2025): 107315, 10.1016/j.ijid.2024.107315. 39613252

[91] E. Diro , K. Ritmeijer , M. Boelaert , et al., “Use of Pentamidine as Secondary Prophylaxis to Prevent Visceral Leishmaniasis Relapse in HIV Infected Patients, the First Twelve Months of a Prospective Cohort Study,” PLoS Neglected Tropical Diseases 9 (2015): e0004087, 10.1371/journal.pntd.0004087. 26431253 PMC4591988

[92] R. Chhajed , P. Dahal , S. Singh‐Phulgenda , et al., “Estimating the Proportion of Relapse Following Treatment of Visceral Leishmaniasis: Meta‐Analysis Using Infectious Diseases Data Observatory (IDDO) Systematic Review,” Lancet Regional Health ‐ Southeast Asia 22 (2024): 100317. 38482151 10.1016/j.lansea.2023.100317 PMC10934318

[93] J. R. Luque‐Ortega , B. G. de la Torre , V. Hornillos , et al., “Defeating Leishmania Resistance to Miltefosine (hexadecylphosphocholine) by Peptide‐Mediated Drug Smuggling: A Proof of Mechanism for Trypanosomatid Chemotherapy,” Journal of Controlled Release 161 (2012): 835–842, 10.1016/j.jconrel.2012.05.023. 22609351

[94] R. Kabra , P. Ingale , and S. Singh , “Computationally Designed Synthetic Peptides for Transporter Proteins Imparts Allostericity in Miltefosine Resistant *L. Major* ,” Biochemical Journal 477 (2020): 2007–2026, 10.1042/BCJ20200176. 32391551

[95] J. M. J. Verhoef , M. Meissner , and T. W. A. Kooij , American Society for Microbiology preprint, 2021, 10.1128/mBio.01409-21

[96] J. Abugri , J. Ayariga , S. S. Sunwiale , et al., Heliyon, (Elsevier Ltd, 2022, 10.1016/j.heliyon.2022.e10390.

[97] D. M. Pitale , G. Kaur , M. Baghel , K. J. Kaur , and C. Shaha , “Halictine‐2 Antimicrobial Peptide Shows Promising Anti‐Parasitic Activity Against Leishmania Spp,” Experimental Parasitology 218 (2020): 107987, 10.1016/j.exppara.2020.107987. 32891601

[98] S. Singh‐Phulgenda , R. Kumar , P. Dahal , et al., “Post‐Kala‐Azar Dermal Leishmaniasis (PKDL) Drug Efficacy Study Landscape: A Systematic Scoping Review of Clinical Trials and Observational Studies,” PLoS Neglected Tropical Diseases 18 (2024): e0011635, 10.1371/journal.pntd.0011635. 38626228 PMC11051605

[99] C. A. Roque‐Borda , Q. Zhang , T. P. T. Nguyen , et al., “Synergistic Combinations of Antimicrobial Peptides and Conventional Antibiotics: A Strategy to Delay Resistance Emergence in WHO Priority Bacteria,” Pharmacological Reviews 78 (2025): 100104. 41389440 10.1016/j.pharmr.2025.100104

[100] Y. Huan , Q. Kong , H. Mou , and H. Yi , “Antimicrobial Peptides: Classification, Design, Application and Research Progress in Multiple Fields,” Frontiers in Microbiology 11 (2020): 582779, 10.3389/fmicb.2020.582779. 33178164 PMC7596191

[101] S. H. Kim , Y.‐H. Min , and M. C. Park , “Antimicrobial Peptides: Current Status, Mechanisms of Action, and Strategies to Overcome Therapeutic Limitations,” Microorganisms 13 (2025): 2574, 10.3390/microorganisms13112574. 41304259 PMC12654255

[102] J. T. C. de Pontes , A. B. Toledo Borges , C. A. Roque‐Borda , and F. R. Pavan , “Antimicrobial Peptides as an Alternative for the Eradication of Bacterial Biofilms of Multi‐Drug Resistant Bacteria,” Pharmaceutics 14 (2022): 642, 10.3390/pharmaceutics14030642. 35336016 PMC8950055

[103] A. Hollmann , M. Martinez , P. Maturana , L. C. Semorile , and P. C. Maffia , “Antimicrobial Peptides: Interaction With Model and Biological Membranes and Synergism With Chemical Antibiotics,” Frontiers in Chemistry 6 (2018): 204, 10.3389/fchem.2018.00204. 29922648 PMC5996110

[104] C. S. Carnero Canales , J. I. Marquez Cazorla , R. M. Marquez Cazorla , et al., “Redefining Therapies for Drug‐Resistant Tuberculosis: Synergistic Effects of Antimicrobial Peptides, Nanotechnology, and Computational Design,” Advanced Healthcare Materials 15 (2026): e03964, 10.1002/adhm.202503964. 41549858 PMC13068362

[105] A. Moretta , C. Scieuzo , A. M. Petrone , et al., “Antimicrobial Peptides: A New Hope in Biomedical and Pharmaceutical Fields,” Frontiers in Cellular and Infection Microbiology 11 (2021): 668632, 10.3389/fcimb.2021.668632. 34195099 PMC8238046

[106] C. H. Chen and T. K. Lu , “Development and Challenges of Antimicrobial Peptides for Therapeutic Applications,” Antibiotics, 9 (2020): 24, 10.3390/antibiotics9010024. 31941022 PMC7168295

[107] J. Zhao , X. Zhang , Y. Liu , Z. Geng , J. Dai , and Y. Guo , “Rational Design and Synthesis of Potent Active Antimicrobial Peptides Based on American Oyster Defensin Analogue A3,” RSC Advances 15 (2025): 19954–19965, 10.1039/D5RA02745D. 40510052 PMC12160004

[108] R. Nordström and M. Malmsten , “Delivery Systems for Antimicrobial Peptides,” Advances in Colloid and Interface Science 242 (2017): 17–34, 10.1016/j.cis.2017.01.005. 28159168

[109] P. Tan , H. Fu , and X. Ma , “Design, Optimization, and Nanotechnology of Antimicrobial Peptides: From Exploration to Applications,” Nano Today 39 (2021): 101229, 10.1016/j.nantod.2021.101229.

[110] L. M. D. G. Primo , C. A. Roque‐Borda , C. S. Carnero Canales , et al., “Antimicrobial Peptides Grafted Onto the Surface of N‐Acetylcysteine‐Chitosan Nanoparticles Can Revitalize Drugs Against Clinical Isolates of Mycobacterium Tuberculosis,” Carbohydrate Polymers 323 (2024): 121449, 10.1016/j.carbpol.2023.121449. 37940311

[111] Z. Yang , S. He , H. Wu , T. Yin , L. Wang , and A. Shan , “Nanostructured Antimicrobial Peptides: Crucial Steps of Overcoming the Bottleneck for Clinics,” Frontiers in Microbiology 12 (2021): 710199, 10.3389/fmicb.2021.710199. 34475862 PMC8406695

[112] A. O. Fadaka , N. R. S. Sibuyi , A. M. Madiehe , and M. Meyer , “Nanotechnology‐Based Delivery Systems for Antimicrobial Peptides,” Pharmaceutics 13 (2021): 1795, 10.3390/pharmaceutics13111795. 34834210 PMC8620809

[113] R. El‐Dirany , H. Shahrour , Z. Dirany , et al., “Activity of Anti‐Microbial Peptides (AMPs) Against Leishmania and Other Parasites: An Overview,” Biomolecules 11 (2021), 984, 10.3390/biom11070984. 34356608 PMC8301979

[114] C. L. Hagemann , A. J. Macedo , and T. Tasca , “Therapeutic Potential of Antimicrobial Peptides Against Pathogenic Protozoa,” Parasitology Research 123 (2024): 122, 10.1007/s00436-024-08133-0. 38311672

[115] C. Proaño‐Bolaños , G. Morán‐Marcillo , N. Espinosa de los Monteros‐Silva , et al., “Bioactivity of Synthetic Peptides From Ecuadorian Frog Skin Secretions Against *Leishmania Mexicana*, *Plasmodium Falciparum*, and *Trypanosoma Cruzi* ,” Microbiology Spectrum 12 (2024), 10.1128/spectrum.03339-23. PMC11302733 39012112

[116] S. M. Robledo , S. Pérez‐Silanes , C. Fernández‐Rubio , et al., “Neglected Zoonotic Diseases: Advances in the Development of Cell‐Penetrating and Antimicrobial Peptides Against Leishmaniosis and Chagas Disease,” Pathogens 12 (2023), 10.3390/pathogens12070939. PMC10383258 37513786

[117] C. S. Carnero Canales , J. I. Marquez Cazorla , R. M. Marquez Cazorla , R. M. Sábio , H. A. Santos , and F. R. Pavan , “Combating Gram‐Negative Infections: The Role of Antimicrobial Peptides and Nanotechnology in Overcoming Antibiotic Resistance,” Materials Today Bio 35 (2025): 102381, 10.1016/j.mtbio.2025.102381. PMC12546895 41142413

[118] A. Mishra , F. Qamar , K. Ashrafi , et al., “Emerging Nanotechnology‐Driven Drug Delivery Solutions for Malaria: Addressing Drug Resistance and Improving Therapeutic Success,” International Journal of Pharmaceutics 670 (2025): 125163, 10.1016/j.ijpharm.2024.125163. 39788401

[119] L. Neves Borgheti‐Cardoso , M. San Anselmo , E. Lantero , et al., “Promising Nanomaterials in the Fight Against Malaria,” Journal of Materials Chemistry. B 8 (2020): 9428–9448, 10.1039/D0TB01398F. 32955067

[120] C. Registre , R. D. O. A. Soares , K. T. S. Rubio , O. D. H. Santos , and S. P. Carneiro , “A Systematic Review of Drug‐Carrying Nanosystems Used in the Treatment of Leishmaniasis,” ACS Infectious Diseases 9 (2023): 423–449, 10.1021/acsinfecdis.2c00632. 36795604

[121] C. Palomino‐Cano , E. Moreno , J. M. Irache , and S. Espuelas , “Targeting and Activation of Macrophages in Leishmaniasis. A Focus on Iron Oxide Nanoparticles,” Frontiers in Immunology 15 (2024), 10.3389/fimmu.2024.1437430. PMC11357945 39211053

[122] S. Keleş , J. Alakbarli , B. Akgül , et al., “Nanotechnology Based Drug Delivery Systems for Malaria,” International Journal of Pharmaceutics 666 (2024): 124746, 10.1016/j.ijpharm.2024.124746. 39321903

[123] P. Prasanna , P. Kumar , S. Kumar , et al., “Current Status of Nanoscale Drug Delivery and the Future of Nano‐Vaccine Development for Leishmaniasis – a Review,” Biomedicine & Pharmacotherapy 141 (2021): 111920, 10.1016/j.biopha.2021.111920. 34328115

[124] Y. Wang , L. Zhang , C. Liu , Y. Luo , and D. Chen , “Peptide‐Mediated Nanocarriers for Targeted Drug Delivery: Developments and Strategies,” Pharmaceutics 16 (2024): 240, 10.3390/pharmaceutics16020240. 38399294 PMC10893007

[125] H. Omidian , L. X. Cubeddu , and R. L. Wilson , “Peptide‐Functionalized Nanomedicine: Advancements in Drug Delivery, Diagnostics, and Biomedical Applications,” Molecules (Basel, Switzerland) 30 (2025): 1572, 10.3390/molecules30071572. 40286158 PMC11990896

[126] J. V. Campos , J. T. C. Pontes , C. S. C. Canales , C. A. Roque‐Borda , and F. R. Pavan , “Advancing Nanotechnology: Targeting Biofilm‐Forming Bacteria With Antimicrobial Peptides,” BME Frontiers 6 (2025), 10.34133/bmef.0104. PMC11876546 40041091

[127] N. Gao , J. Sun , X. Li , et al., “Overcoming Delivery Challenges of Antimicrobial Peptides for Clinical Translation: From Nanocarriers to Molecular Modifications,” Drug Resistance Updates 83 (2025): 101289, 10.1016/j.drup.2025.101289. 40795792

[128] A. M. Jankowski , M. A. Ensign , and K. Maisel , “Cell‐Penetrating Peptides as Facilitators of Cargo‐Specific Nanocarrier‐Based Drug Delivery,” Nanoscale 17 (2025): 20006–20019, 10.1039/D5NR00617A. 40856125 PMC12379189

[129] J. Wu , S. Roesger , N. Jones , C.‐M. J. Hu , and S.‐D. Li , “Cell‐Penetrating Peptides for Transmucosal Delivery of Proteins,” Journal of Controlled Release 366 (2024): 864–878, 10.1016/j.jconrel.2024.01.038. 38272399

[130] S. Chatterjee , E. Kon , P. Sharma , and D. Peer , “Endosomal Escape: A Bottleneck for LNP‐Mediated Therapeutics,” Proceedings of the National Academy of Sciences 121 (2024), 10.1073/pnas.2307800120. PMC10945858 38437552

[131] J. Soukar , N. A. Peppas , and A. K. Gaharwar , “Organelle‐Targeting Nanoparticles,” Advanced Science 12 (2025), 10.1002/advs.202411720. PMC11831507 39806939

[132] A. N. Shirazi , R. Vadlapatla , A. Koomer , A. Nguyen , V. Khoury , and K. Parang , “Peptide‐Based Inorganic Nanoparticles as Efficient Intracellular Delivery Systems,” Pharmaceutics 17 (2025): 1123, 10.3390/pharmaceutics17091123. 41012460 PMC12472943

[133] T. Zhang , X. Luo , K. Xu , and W. Zhong , “Peptide‐Containing Nanoformulations: Skin Barrier Penetration and Activity Contribution,” Advanced Drug Delivery Reviews 203 (2023): 115139, 10.1016/j.addr.2023.115139. 37951358

[134] H. Zhao , J. Sun , Y. Cheng , S. Nie , and W. Li , “Advances in Peptide/Polymer Antimicrobial Assemblies,” Journal of Materials Chemistry B 13 (2025): 1518–1530, 10.1039/D4TB02144D. 39714335

[135] K.‐C. Mei , N. Thota , P.‐S. Wei , B. Yi , E. E. Bonacquisti , and J. Nguyen , “Calreticulin P‐Domain‐Derived Eat‐Me Peptides for Enhancing Liposomal Uptake in Dendritic Cells,” International Journal of Pharmaceutics 653 (2024): 123844, 10.1016/j.ijpharm.2024.123844. 38272193 PMC10994729

[136] M.‐A. Jourdain , A. Dupont , N. Lautram , and J. Eyer , “Investigating the Functionalization of Liposomes With NFL‐TBS. 40‐63 Peptide as a Promising Drug Delivery System,” International Journal of Pharmaceutics 652 (2024): 123805, 10.1016/j.ijpharm.2024.123805. 38237710

[137] Y. Yang , Y. Chu , C. Li , et al., “Brain‐Targeted Drug Delivery by In Vivo Functionalized Liposome With Stable D‐Peptide Ligand,” Journal of Controlled Release 373 (2024): 240–251, 10.1016/j.jconrel.2024.07.014. 38977135

[138] M. Shi and K. J. McHugh , “Strategies for Overcoming Protein and Peptide Instability in Biodegradable Drug Delivery Systems,” Advanced Drug Delivery Reviews 199 (2023): 114904, 10.1016/j.addr.2023.114904. 37263542 PMC10526705

[139] J. J. Rodriguez‐Cruz , F. A. Chapa‐Villarreal , I. Duggal , and N. A. Peppas , “Advanced Rational Design of Polymeric Nanoparticles for the Controlled Delivery of Biologics,” Journal of Controlled Release 384 (2025): 113940, 10.1016/j.jconrel.2025.113940. 40484283

[140] M. Elsabahy , Y. Song , N. G. Eissa , S. Khan , M. A. Hamad , and K. L. Wooley , “Morphologic Design of Sugar‐Based Polymer Nanoparticles for Delivery of Antidiabetic Peptides,” Journal of Controlled Release 334 (2021): 1–10, 10.1016/j.jconrel.2021.04.006. 33845056

[141] P. Joyce , C. J. Allen , M. J. Alonso , et al., “A Translational Framework to DELIVER Nanomedicines to the Clinic,” Nature Nanotechnology 19 (2024): 1597–1611, 10.1038/s41565-024-01754-7. 39242807

[142] O. S. Thomas and W. Weber , “Overcoming Physiological Barriers to Nanoparticle Delivery—Are We There Yet?,” Frontiers in Bioengineering and Biotechnology 7 (2019): 415, 10.3389/fbioe.2019.00415. 31921819 PMC6928054

[143] Y. Shen , H. Gwak , and B. Han , “Advanced Manufacturing of Nanoparticle Formulations of Drugs and Biologics Using Microfluidics,” Analyst 149 (2024): 614–637, 10.1039/D3AN01739G. 38083968 PMC10842755

[144] V. Sebastian , “Toward Continuous Production of High‐Quality Nanomaterials Using Microfluidics: Nanoengineering the Shape, Structure and Chemical Composition,” Nanoscale 14 (2022): 4411–4447, 10.1039/D1NR06342A. 35274121

[145] F. Fu , D. Crespy , K. Landfester , and S. Jiang , “ *In Situ* Characterization Techniques of Protein Corona Around Nanomaterials,” Chemical Society Reviews 53 (2024): 10827–10851, 10.1039/D4CS00507D. 39291461

[146] S. Li , C. Cortez‐Jugo , Y. Ju , and F. Caruso , “Approaching Two Decades: Biomolecular Coronas and Bio–Nano Interactions,” ACS Nano 18 (2024): 33257–33263, 10.1021/acsnano.4c13214. 39602410

[147] A. Canchola , K. Li , K. Chen , et al., “Meta‐Analysis and Machine Learning Prediction of Protein Corona Composition Across Nanoparticle Systems in Biological Media,” ACS Nano 19 (2025): 37633–37650, 10.1021/acsnano.5c08608. 41031442 PMC12593346

[148] W.‐C. Chou and Z. Lin , “Impact of Protein Coronas on Nanoparticle Interactions With Tissues and Targeted Delivery,” Current Opinion in Biotechnology 85 (2024): 103046, 10.1016/j.copbio.2023.103046. 38103519 PMC11000521

[149] D. Kelle , K. R. Speth , M. Martínez‐Negro , V. Mailänder , K. Landfester , and B. Iyisan , “Effect of Protein Corona on Drug Release Behavior of PLGA Nanoparticles,” European Journal of Pharmaceutics and Biopharmaceutics 207 (2025): 114611, 10.1016/j.ejpb.2024.114611. 39674519

[150] I. V. Zelepukin , K. G. Shevchenko , and S. M. Deyev , “Rediscovery of Mononuclear Phagocyte System Blockade for Nanoparticle Drug Delivery,” Nature Communications 15 (2024): 4366, 10.1038/s41467-024-48838-5. PMC11111695 38777821

[151] K. Hulugalla , O. Shofolawe‐Bakare , V. B. Toragall , et al., “Glycopolymeric Nanoparticles Enrich Less Immunogenic Protein Coronas, Reduce Mononuclear Phagocyte Clearance, and Improve Tumor Delivery Compared to PEGylated Nanoparticles,” ACS Nano 18 (2024): 30540–30560, 10.1021/acsnano.4c08922. 39436672 PMC12045476

[152] Y. Miao , L. Li , Y. Wang , et al., “Regulating Protein Corona on Nanovesicles by Glycosylated Polyhydroxy Polymer Modification for Efficient Drug Delivery,” Nature Communications 15 (2024): 1159, 10.1038/s41467-024-45254-7. PMC10850157 38326312

[153] Y. Zhou , X. Wang , X. Tian , et al., “Stealth Missiles With Precision Guidance: A Novel Multifunctional Nano‐Drug Delivery System Based on Biomimetic Cell Membrane Coating Technology,” Materials Today Bio 33 (2025): 101922, 10.1016/j.mtbio.2025.101922. PMC12173624 40528836

[154] J. Feng , D. He , J. Chen , et al., “Cell Membrane Biomimetic Nanoplatforms: A New Strategy for Immune Escape and Precision Targeted Therapy,” Materials Today Bio 35 (2025): 102343, 10.1016/j.mtbio.2025.102343. PMC12506577 41069689

[155] S. Đorđević , M. M. Gonzalez , I. Conejos‐Sánchez , et al., “Current Hurdles to the Translation of Nanomedicines From Bench to the Clinic,” Drug Delivery and Translational Research 12 (2022): 500–525, 10.1007/s13346-021-01024-2. 34302274 PMC8300981

[156] D. Zhao , J. Liang , X. Huang , S. Shen , and J. Wang , “Organoids Technology for Advancing the Clinical Translation of Cancer Nanomedicine,” WIREs Nanomedicine and Nanobiotechnology 15 (2023): e1892, 10.1002/wnan.1892. 37088100

[157] Y. Hou , R. Wu , Y. Zhou , et al., “Macrophage Membrane‐Coated Nanoparticles in Inflammatory Diseases: From Bioinspired Design to Translational Potential,” Journal of Nanobiotechnology 24 (2026): 37, 10.1186/s12951-025-03921-x. PMC12801971 41366426

[158] A. Kumar , R. Tiwari , S. Kumari , et al., “Cyclodextrin‐AmB‐IL‐10 Antagonist Peptide Nanoparticles Treat Leishmaniasis More Effectively Than Conventional AmB,” Nanomedicine 20 (2025): 2495–2509, 10.1080/17435889.2025.2552099. 40878891 PMC12505504

[159] C. Wang , C. Li , R. Zhang , and L. Huang , “Macrophage Membrane‐Coated Nanoparticles for the Treatment of Infectious Diseases,” Biomedical Materials 19 (2024): 042003, 10.1088/1748-605X/ad4aaa. 38740051

[160] J. Wang , Y. Li , R. M. Reguera , et al., “Lymphatic‐Targeted Amphotericin B Nanocrystals Delivered Using Microarray Patches Applied to Cutaneous Leishmaniasis,” Journal of Controlled Release 386 (2025): 114115, 10.1016/j.jconrel.2025.114115. 40796014

[161] S. Sundar , K. Pandey , D. Mondal , et al., “A Phase II, Non‐Comparative Randomised Trial of Two Treatments Involving Liposomal Amphotericin B and Miltefosine for Post‐Kala‐Azar Dermal Leishmaniasis in India and Bangladesh,” PLoS Neglected Tropical Diseases 18 (2024): e0012242, 10.1371/journal.pntd.0012242. 38900786 PMC11189210

[162] D. Cosco , F. Bruno , G. Castelli , et al., “Meglumine Antimoniate‐Loaded Aqueous‐Core PLA Nanocapsules: Old Drug, New Formulation Against *Leishmania* ‐Related Diseases,” Macromolecular Bioscience 21 (2021): 2100046, 10.1002/mabi.202100046. 34117834

[163] Y. Avalos‐Padilla and X. Fernàndez‐Busquets , “Nanotherapeutics Against Malaria: A Decade of Advancements in Experimental Models,” WIREs Nanomedicine and Nanobiotechnology 16 (2024): e1943, 10.1002/wnan.1943. 38426407

[164] N. Dridi , Z. Jin , W. Perng , and H. Mattoussi , “Probing Protein Corona Formation Around Gold Nanoparticles: Effects of Surface Coating,” ACS Nano 18 (2024): 8649–8662, 10.1021/acsnano.3c08005. 38471029

[165] W. Xiao , W. Jiang , Z. Chen , et al., “Advance in Peptide‐Based Drug Development: Delivery Platforms, Therapeutics and Vaccines,” Signal Transduction and Targeted Therapy 10 (2025): 74, 10.1038/s41392-024-02107-5. 40038239 PMC11880366

[166] L. Chai , X. Niu , X. Li , et al., “Oral Nanoliposomes Functionalized With cRGD and Polydopamine for Enhanced Antimalarial Efficacy of Disulfide Bond‐Modified Dihydroartemisinin Prodrug,” Drug Delivery 32 (2025): 2554079, 10.1080/10717544.2025.2554079. 41078131 PMC12519588

[167] S. Soussi , R. Essid , I. Karkouch , et al., “Effect of Lipopeptide‐Loaded Chitosan Nanoparticles on Candida Albicans Adhesion and on the Growth of Leishmania Major,” Applied Biochemistry and Biotechnology 193 (2021): 3732–3752, 10.1007/s12010-021-03621-w. 34398423

[168] S. Akhzari , S. Nabian , and M. Taheri , “In Vitro Evaluation of Activatable Melittin Encapsulated in Liposome and Albumin Nanoparticles Against Leishmania,” Veterinary Research Forum 17 (2026): 127–133, 10.30466/vrf.2025.2047654.4572. 42327699 PMC13276830

[169] P. M. Daftarian , G. W. Stone , L. Kovalski , et al., “A Targeted and Adjuvanted Nanocarrier Lowers the Effective Dose of Liposomal Amphotericin B and Enhances Adaptive Immunity in Murine Cutaneous Leishmaniasis,” Journal of Infectious Diseases 208 (2013): 1914–1922, 10.1093/infdis/jit378. 23901083 PMC3814840

[170] U. Ornellas‐Garcia , P. Cuervo , and F. L. Ribeiro‐Gomes , “Malaria and Leishmaniasis: Updates on Co‐Infection,” Frontiers in Immunology 14 (2023): 1122411, 10.3389/fimmu.2023.1122411. 36895563 PMC9989157

[171] M. Hassan , A. Omar , I. Mohamed , B. Garba , M. M. A. Fuje , and S. O. Salad , “A Late Diagnosis of Visceral Leishmaniasis Using Tru‐Cut Biopsy of the Spleen and Malaria Co‐Infection – A Diagnostic Challenge: A Case Report in Somalia,” Infection and Drug Resistance 16 (2023): 6513–6519, 10.2147/IDR.S420832. 37809037 PMC10557959

[172] A. Maqsood , M. S. Farid , M. H. Khan , and M. Grzegorzek , “Deep Malaria Parasite Detection in Thin Blood Smear Microscopic Images,” Applied Sciences 11 (2021): 2284, 10.3390/app11052284.

[173] E. F. Santos , R. T. Daltro , C. G. Regis‐Silva , et al., “Assessment of Cross‐Reactivity of Chimeric Trypanosoma Cruzi Antigens With Crithidia sp. LVH‐60A: Implications for Accurate Diagnostics,” Diagnostics 13 (2023): 3470, 10.3390/diagnostics13223470. 37998606 PMC10670697

[174] M. M. Bautista‐Gomez , J. Doerfler , and M. del Mar Castro , “Barriers to Cutaneous Leishmaniasis Care Faced by Indigenous Communities of Rural Areas in Colombia: A Qualitative Study,” BMC Infectious Diseases 22 (2022): 302, 10.1186/s12879-022-07204-w. 35351012 PMC8962053

[175] X. Wang , J. Zhou , and H. Wang , “Bioreceptors as the Key Components for Electrochemical Biosensing in Medicine,” Cell Reports Physical Science 5 (2024): 101801, 10.1016/j.xcrp.2024.101801.

[176] M. A. Darwish , W. Abd‐Elaziem , A. Elsheikh , and A. A. Zayed , “Advancements in Nanomaterials for Nanosensors: A Comprehensive Review,” Nanoscale Advances 6 (2024): 4015–4046, 10.1039/D4NA00214H. 39114135 PMC11304082

[177] K. J. Land , D. I. Boeras , X.‐S. Chen , A. R. Ramsay , and R. W. Peeling , “REASSURED Diagnostics to Inform Disease Control Strategies, Strengthen Health Systems and Improve Patient Outcomes,” Nature Microbiology 4 (2019): 46–54, 10.1038/s41564-018-0295-3. PMC7097043 30546093

[178] P. M. Bossuyt , J. B. Reitsma , D. E. Bruns , et al., “STARD 2015: An Updated List of Essential Items for Reporting Diagnostic Accuracy Studies,” BMJ 351 (2015): h5527, 10.1136/bmj.h5527. 26511519 PMC4623764

[179] J. N. Cruz , A. P. da Silva Souza Filho , M. E. C. Oliveira , D. S. Pereira , and M. S. de , Oliveira in Applications of Nanobiotechnology for Neglected Tropical Diseases (Elsevier, 2021). 107–116.

[180] L. Fritea , F. Banica , T. Costea , et al., “Metal Nanoparticles and Carbon‐Based Nanomaterials for Improved Performances of Electrochemical (Bio)Sensors With Biomedical Applications,” Materials 14 (2021): 6319, 10.3390/ma14216319. 34771844 PMC8585379

[181] S. M. Feleke , E. N. Reichert , H. Mohammed , et al., “Plasmodium Falciparum Is Evolving to Escape Malaria Rapid Diagnostic Tests in Ethiopia,” Nature Microbiology 6 (2021): 1289–1299, 10.1038/s41564-021-00962-4. PMC8478644 34580442

[182] A. Patel , J. Patel , R. Patel , and V Patidar , Infectious Diseases Drug Delivery Systems (Springer International Publishing, 2023): 77–99.

[183] V. Thirumalaiswamy , C. V. Vaishali , S. Sundararaju , C. M. Ramakritinan , M. Thillaichidambaram , and F. Quero , Nanoscience and Nanotechnology for Smart Prevention, Diagnostics and Therapeutics (Wiley, 2024), 143–181.

[184] S. Sadr , A. Hajjafari , A. Sazmand , et al., “Nanobiosensors for Revolutionizing Parasitic Infections Diagnosis: A Critical Review to Improve Global Health With an Update on Future Challenges Prospect,” European Journal of Medical Research 30 (2025): 484, 10.1186/s40001-025-02685-2. 40524269 PMC12168311

[185] G. Figueroa‐Miranda , S. Chen , M. Neis , et al., “Multi‐Target Electrochemical Malaria Aptasensor on Flexible Multielectrode Arrays for Detection in Malaria Parasite Blood Samples,” Sensors and Actuators B: Chemical 349 (2021): 130812, 10.1016/j.snb.2021.130812.

[186] A. Minopoli , B. Della Ventura , B. Lenyk , et al., “Ultrasensitive Antibody‐Aptamer Plasmonic Biosensor for Malaria Biomarker Detection in Whole Blood,” Nature Communications 11 (2020): 6134, 10.1038/s41467-020-19755-0. PMC7708447 33262332

[187] E. Dueñas , J. A. Nakamoto , L. Cabrera‐Sosa , et al., “Novel CRISPR‐Based Detection of Leishmania Species,” Frontiers in Microbiology 13 (2022), 10.3389/fmicb.2022.958693. PMC9520526 36187950

[188] A. J. Caraballo‐Guzmán , J. D. Ospina‐Villa , A. P. Cuesta‐Caicedo , and M. M. Sánchez‐Jiménez , “Immunoproteomics Characterization of *Leishmania Panamensis* Proteins for Potential Clinical Diagnosis of Mucosal Leishmaniasis,” Parasite Immunology 43 (2021): e12824, 10.1111/pim.12824. 33484577

[189] J. D. Ospina‐Villa , C. López‐Camarillo , C. A. Castañón‐Sánchez , J. Soto‐Sánchez , E. Ramírez‐Moreno , and L. A. Marchat , “Advances on Aptamers Against Protozoan Parasites,” Genes 9 (2018): 584, 10.3390/genes9120584. 30487456 PMC6316487

[190] M. R. Gedda , P. Madhukar , A. Shukla , et al., “Nanodiagnostics in Leishmaniasis: A New Frontiers for Early Elimination,” WIREs Nanomedicine and Nanobiotechnology 13 (2021): e1675, 10.1002/wnan.1675. 33142369 PMC7897294

[191] G. Can , A. Chouihi , M. F. Diouani , and Ü. Anık , “Rapid and Practical Colorimetric Biosensor for Leishmaniasis Diseases,” Diagnostic Microbiology and Infectious Disease 109 (2024): 116352, 10.1016/j.diagmicrobio.2024.116352. 38768547

[192] B. Perk , Y. Tepeli Büyüksünetçi , S. Bachraoui Bouzaien , M. F. Diouani , and Ü. Anik , “Fabrication of Metal–organic Framework Based Electrochemical Leishmania Immunosensor,” Microchemical Journal 192 (2023): 108958, 10.1016/j.microc.2023.108958.

[193] J. D. Ospina‐Villa , A. Cisneros‐Sarabia , M. M. Sánchez‐Jiménez , and L. A. Marchat , “Current Advances in the Development of Diagnostic Tests Based on Aptamers in Parasitology: A Systematic Review,” Pharmaceutics 12 (2020): 1046, 10.3390/pharmaceutics12111046. 33142793 PMC7693570

[194] S. Jain , W. Santana , S. S. Dolabella , A. L. S. Santos , E. B. Souto , and P. Severino , Advances in Medical Imaging, Detection, and Diagnosis (Jenny Stanford Publishing, 2023), 783–811.

[195] N. E. Brosseau , I. Vallée , A. Mayer‐Scholl , M. Ndao , and G. Karadjian , “Aptamer‐Based Technologies for Parasite Detection,” Sensors 23 (2023): 562, 10.3390/s23020562. 36679358 PMC9867382

[196] M. Sheraz , X.‐F. Sun , Y. Wang , J. Chen , and L. Sun , “Recent Developments in Aptamer‐Based Sensors for Diagnostics,” Sensors 24 (2024): 7432, 10.3390/s24237432. 39685966 PMC11644826

[197] A. Muslihati , N. L. W. Septiani , G. Gumilar , N. Nugraha , H. S. Wasisto , and B. Yuliarto , “Peptide‐Based Flavivirus Biosensors: From Cell Structure to Virological and Serological Detection Methods,” ACS Biomaterials Science & Engineering 10 (2024): 2041–2061, 10.1021/acsbiomaterials.3c01965. 38526408

[198] S. Premchanth Jyothi , S. Sadanandan , and R. Rajamani , “A Critical Review on the Identification of Pathogens by Employing Peptide‐Based Electrochemical Biosensors,” Critical Reviews in Analytical Chemistry 56 (2024): 1–14. 39163555 10.1080/10408347.2024.2390551

[199] M. G. Sande , J. L. Rodrigues , D. Ferreira , C. J. Silva , and L. R. Rodrigues , “Novel Biorecognition Elements Against Pathogens in the Design of State‐of‐the‐Art Diagnostics,” Biosensors (Basel) 11 (2021): 418, 10.3390/bios11110418. 34821636 PMC8615483

[200] G. Tegegn , N. Gnanasekaren , E. Gadisa , et al., “Comparative Assessment of Microscopy, Malaria Rapid Diagnostic Test and Polymerase Chain Reaction as Malaria Diagnostic Tools in Adama Woreda, East Shoa Zone of Ethiopia: A Cross‐Sectional Study,” BMC Infectious Diseases 24 (2024): 1363, 10.1186/s12879-024-10173-x. 39609673 PMC11603952

[201] E. R. Adams , M. A. Gomez , L. Scheske , et al., “Sensitive Diagnosis of Cutaneous Leishmaniasis by Lesion Swab Sampling Coupled to qPCR,” Parasitology 141 (2014): 1891–1897, 10.1017/S0031182014001280. 25111885 PMC4654403

[202] M. Koerselman , L. C. M. Morshuis , and M. Karperien , “The use of Peptides, Aptamers, and Variable Domains of Heavy Chain Only Antibodies in Tissue Engineering and Regenerative Medicine,” Acta Biomaterialia 170 (2023): 1–14, 10.1016/j.actbio.2023.07.045. 37517622

[203] S. Ni , Z. Zhuo , Y. Pan , et al., “Recent Progress in Aptamer Discoveries and Modifications for Therapeutic Applications,” ACS Applied Materials & Interfaces 13 (2020): 9500–9519, 10.1021/acsami.0c05750. 32603135

[204] C. S. Carnero Canales , C. A. Roque‐Borda , J. I. M. Cazorla , et al., “Forging a New Frontier: Antimicrobial Peptides and Nanotechnology Converging to Conquer Gastrointestinal Pathogens,” Small 21 (2025): 2501431, 10.1002/smll.202501431. 40384187 PMC12232244

[205] C. S. Carnero Canales , J. I. Marquez Cazorla , R. M. Marquez Cazorla , et al., “Breaking Barriers: The Potential of Nanosystems in Antituberculosis Therapy,” Bioactive Materials 39 (2024): 106–134, 10.1016/j.bioactmat.2024.05.013. 38783925 PMC11112550

[206] K. Pandiyaraj , R. A. Elkaffas , M. I. H. Mohideen , and S. Eissa , “Graphene Oxide/Cu–MOF‐Based Electrochemical Immunosensor for the Simultaneous Detection of Mycoplasma Pneumoniae and Legionella Pneumophila Antigens in Water,” Scientific Reports 14 (2024): 17172, 10.1038/s41598-024-68231-y. 39060466 PMC11282068

[207] C. Herman , C. S. Huber , S. Jones , et al., “Multiplex Malaria Antigen Detection by Bead‐Based Assay and Molecular Confirmation by PCR Shows No Evidence of Pfhrp2 and Pfhrp3 Deletion in Haiti,” Malaria Journal 18 (2019): 380, 10.1186/s12936-019-3010-9. 31775743 PMC6882344

[208] R. Alvarado , L. L. van den Hoogen , N. C. Iriemenam , et al., “Considerations for Quality Assurance of Multiplex Malaria Antigen Detection Assays With Large Sample Sets,” Scientific Reports 11 (2021): 13248, 10.1038/s41598-021-92723-w. 34168264 PMC8225651

[209] S. Jain , S. Gupta , S. Patiyal , and G. P. S. Raghava , “THPdb2: Compilation of FDA Approved Therapeutic Peptides and Proteins,” Drug Discovery Today 29 (2024): 104047, 10.1016/j.drudis.2024.104047. 38830503

[210] L. Cresti , G. Cappello , and A. Pini , “Antimicrobial Peptides Towards Clinical Application—a Long History to Be Concluded,” International Journal of Molecular Sciences 25 (2024): 4870, 10.3390/ijms25094870. 38732089 PMC11084544

[211] T. N. der Kolk , T. P. Buters , L. Krouwels , et al., “Topical Antimicrobial Peptide Omiganan Recovers Cutaneous Dysbiosis but Does Not Improve Clinical Symptoms in Patients With Mild to Moderate Atopic Dermatitis in a Phase 2 randomized Controlled Trial,” Journal of the American Academy of Dermatology 86 (2022): 854–862, 10.1016/j.jaad.2020.08.132. 33010325

[212] M. Hernández‐García , R. Barbero‐Herranz , N. Bastón‐Paz , et al., “Unravelling the Mechanisms Causing Murepavadin Resistance in Pseudomonas Aeruginosa: Lipopolysaccharide Alterations and Its Consequences,” Frontiers in Cellular and Infection Microbiology 14 (2024): 1446626, 10.3389/fcimb.2024.1446626. 39711784 PMC11659217

[213] Q. Li , W. Chao , and L. Qiu , “Therapeutic Peptides: Chemical Strategies Fortify Peptides for Enhanced Disease Treatment Efficacy,” Amino Acids 57 (2025): 25, 10.1007/s00726-025-03454-5. 40338379 PMC12062087

[214] S. B. H. Kent , “Fundamental Aspects of SPPS and Green Chemical Peptide Synthesis,” Journal of Peptide Science 31 (2025), 10.1002/psc.70013. PMC11985259 40210223

[215] A. K. Kuril , K. Saravanan , and P. K. Subbappa , “Analytical Considerations for Characterization of Generic Peptide Product: A Regulatory Insight,” Analytical Biochemistry 694 (2024): 115633, 10.1016/j.ab.2024.115633. 39089363

[216] M. del M. Castro , A. C. Erber , B. Arana , et al., “Involving Patients in Drug Development for Neglected Tropical Diseases (NTDs): A Qualitative Study Exploring and Incorporating Preferences of Patients With Cutaneous Leishmaniasis Into Target Product Profile Development,” PLoS Neglected Tropical Diseases 18 (2024): e0011975, 10.1371/journal.pntd.0011975. 38381805 PMC10965092

[217] Z. Sun , X. Wang , S. Kuang , J. Song , and C. de la Fuente‐Nunez , “From Generation to Validation: Deep Generative Models for Antimicrobial Peptide Discovery,” Current Opinion in Chemical Biology 93 (2026): 102685, 10.1016/j.cbpa.2026.102685. 42054758

[218] Z. Lai , X. Yuan , H. Chen , Y. Zhu , N. Dong , and A. Shan , “Strategies Employed in the Design of Antimicrobial Peptides With Enhanced Proteolytic Stability,” Biotechnology Advances 59 (2022): 107962, 10.1016/j.biotechadv.2022.107962. 35452776

[219] Y. You , H. Liu , Y. Zhu , and H. Zheng , “Rational Design of Stapled Antimicrobial Peptides,” Amino Acids 55 (2023): 421–442, 10.1007/s00726-023-03245-w. 36781451

[220] Y. Li , M. Wu , Y. Fu , et al., “Therapeutic Stapled Peptides: Efficacy and Molecular Targets,” Pharmacological Research 203 (2024): 107137, 10.1016/j.phrs.2024.107137. 38522761

[221] R. Bellavita , S. Braccia , S. Galdiero , and A. Falanga , “Glycosylation and Lipidation Strategies: Approaches for Improving Antimicrobial Peptide Efficacy,” Pharmaceuticals 16 (2023): 439, 10.3390/ph16030439. 36986538 PMC10059750

[222] W. Yu , M. Zhao , X. Guo , et al., “Proteolytic‐Resistant Self‐Assembling Peptide Nanofibers Combat Specific Bacterial Infections via Trap and Kill,” Science Advances 11 (2025): eadx0153, 10.1126/sciadv.adx0153. 40680130 PMC12273793

[223] D. Campoccia , G. Bottau , A. De Donno , et al., “Assessing Cytotoxicity, Proteolytic Stability, and Selectivity of Antimicrobial Peptides: Implications for Orthopedic Applications,” International Journal of Molecular Sciences 25 (2024): 13241, 10.3390/ijms252413241. 39769006 PMC11678430

[224] E. P. Fletcher , M. Sahre , Y. Y. Hon , et al., “Impact of Organ Impairment on the Pharmacokinetics of Therapeutic Peptides and Proteins,” AAPS Journal 25 (2023): 54, 10.1208/s12248-023-00819-0. 37231199

[225] M. Park , F. M. Dannheim , M. A. Papworth , et al., “Boosting Peptide Half‐Life: Enabling Efficient Generation of Fc‐Peptide Conjugates,” Chemical Science 17 (2026): 13454–13464, 10.1039/D6SC02646J. 42266899 PMC13244299

[226] J. Höppner , H. Noda , A. K. Anitha , et al., “Prolonging Parathyroid Hormone Analog Action In Vitro and In Vivo Through Peptide Lipidation,” Nature Communications 16 (2025): 4487, 10.1038/s41467-025-59665-7. PMC12078793 40368898

[227] G. Chen , W. Kang , W. Li , S. Chen , and Y. Gao , “Oral Delivery of Protein and Peptide Drugs: From Non‐Specific Formulation Approaches to Intestinal Cell Targeting Strategies,” Theranostics 12 (2022): 1419–1439, 10.7150/thno.61747. 35154498 PMC8771547

[228] G. Chen , “Advances in the Oral Delivery of Protein and Peptide Drugs,” Pharmaceutics 17 (2025): 616, 10.3390/pharmaceutics17050616. 40430907 PMC12114803

[229] J. Achan , A. Barry , D. Leroy , et al., “Defining the Next Generation of Severe Malaria Treatment: A Target Product Profile,” Malaria Journal 23 (2024): 174, 10.1186/s12936-024-04986-z. 38835069 PMC11151482

[230] S. Braillard , M. Keenan , K. J. Breese , et al., “DNDI‐6174 Is a Preclinical Candidate for Visceral Leishmaniasis that Targets the Cytochrome bc_1_ ,” Science Translational Medicine 15 (2023): eadh9902, 10.1126/scitranslmed.adh9902. 38091406 PMC7615677

[231] A. S. De Groot , B. J. Roberts , A. Mattei , S. Lelias , C. Boyle , and W. D. Martin , “Immunogenicity Risk Assessment of Synthetic Peptide Drugs and Their Impurities,” Drug Discovery Today 28 (2023): 103714, 10.1016/j.drudis.2023.103714. 37467878

[232] S. P. Mihindukulasooriya , Z. Wang , S. Li , et al., “Anti‐PEG Antibodies in Nanomedicine: Mechanisms, Risks, and Opportunities,” Advanced Drug Delivery Reviews 236 (2026): 115925, 10.1016/j.addr.2026.115925. 42362057

[233] M. E. Senti , C. A. de Jongh , K. Dijkxhoorn , et al., “Anti‐PEG Antibodies Compromise the Integrity of PEGylated Lipid‐Based Nanoparticles via Complement,” Journal of Controlled Release 341 (2022): 475–486, 10.1016/j.jconrel.2021.11.042. 34890719

[234] J. Ren , N. Andrikopoulos , K. Velonia , et al., “Chemical and Biophysical Signatures of the Protein Corona in Nanomedicine,” Journal of the American Chemical Society 144 (2022): 9184–9205, 10.1021/jacs.2c02277. 35536591

[235] Z. Jiang , Y. Chu , and C. Zhan , “Protein Corona: Challenges and Opportunities for Targeted Delivery of Nanomedicines,” Expert Opinion on Drug Delivery 19 (2022): 833–846, 10.1080/17425247.2022.2093854. 35738018

[236] I. Kekessie , K. Wegner , I. Martinez , et al., “Process Mass Intensity (PMI): A Holistic Analysis of Current Peptide Manufacturing Processes Informs Sustainability in Peptide Synthesis,” Journal of Organic Chemistry 89 (2024): 4261–4282, 10.1021/acs.joc.3c01494. 38508870 PMC11002941

[237] J. M. Collins , S. K. Singh , T. A. White , et al., “Total Wash Elimination for Solid Phase Peptide Synthesis,” Nature Communications 14 (2023): 8168, 10.1038/s41467-023-44074-5. PMC10710472 38071224

[238] A. Sharma , A. Kumar , B. G. de la Torre , and F. Albericio , “Liquid‐Phase Peptide Synthesis (LPPS): A Third Wave for the Preparation of Peptides,” Chemical Reviews 122 (2022): 13516–13546, 10.1021/acs.chemrev.2c00132. 35816287

[239] I. Seder , T. Zheng , J. Zhang , et al., “A Scalable Microfluidic Platform for Nanoparticle Formulation: For Exploratory‐ and Industrial‐Level Scales,” Nano Letters 24 (2024): 5132–5138, 10.1021/acs.nanolett.3c05057. 38588326

[240] A. R. Hanna , S. J. Shepherd , G. A. Datto , et al., “Automated and Parallelized Microfluidic Generation of Large and Precisely Defined Lipid Nanoparticle Libraries,” ACS Nano 20 (2026): 772–789, 10.1021/acsnano.5c15613. 41453807

[241] R. Jin , X. Fu , Y. Pu , et al., “Clinical Translational Barriers Against Nanoparticle‐Based Imaging Agents,” Advanced Drug Delivery Reviews 191 (2022): 114587, 10.1016/j.addr.2022.114587. 36309148

[242] D. Badgujar , S. T. Paritala , S. Matre , and N. Sharma , “Enantiomeric Purity of Synthetic Therapeutic Peptides: A Review,” Chirality 36 (2024): e23652, 10.1002/chir.v36.3. 38448043

[243] D. McCarthy , Y. Han , K. Carrick , et al., “Reference Standards to Support Quality of Synthetic Peptide Therapeutics,” Pharmaceutical Research 40 (2023): 1317–1328, 10.1007/s11095-023-03493-1. 36949371 PMC10338602

[244] K. Sharma , K. K. Sharma , A. Sharma , and R. Jain , “Peptide‐Based Drug Discovery: Current Status and Recent Advances,” Drug Discovery Today 28 (2023): 103464, 10.1016/j.drudis.2022.103464. 36481586

[245] M. Liu , D. Svirskis , T. Proft , et al., “Progress in Peptide and Protein Therapeutics: Challenges and Strategies,” Acta Pharmaceutica Sinica B 15 (2025): 6342–6381, 10.1016/j.apsb.2025.10.026. 41477339 PMC12750180

[246] F. Manca , G. Ciminata , E. Grieve , J. Reboud , J. Cooper , and E. McIntosh , “Cost‐Effectiveness of Sentinel Screening of Endemic Diseases Alongside Malaria Diagnosis: A Case Study in Schistosomiasis,” PLoS Neglected Tropical Diseases 18 (2024): e0012339, 10.1371/journal.pntd.0012339. 39074148 PMC11309411

[247] N. Docherty , D. Macdonald , A. Gordon , et al., “Maximising the Translation Potential of Electrochemical Biosensors,” Chemical Communications 61 (2025): 13359–13377, 10.1039/D5CC02322J. 40814868 PMC12355650

[248] F. Wan , F. Wong , J. J. Collins , and C. de la Fuente‐Nunez , “Machine Learning for Antimicrobial Peptide Identification and Design,” Nature Reviews Bioengineering 2 (2024): 392–407, 10.1038/s44222-024-00152-x. PMC11756916 39850516

[249] B. Mishra , A. Basu , F. Shehadeh , et al., “Antimicrobial Peptide Developed With Machine Learning Sequence Optimization Targets Drug Resistant Staphylococcus Aureus in Mice,” Journal of Clinical Investigation 135 (2025): e185430, 10.1172/JCI185430. 40261713 PMC12165799

[250] Q. Kong , Y. Zhao , H. Gong , et al., “AI Agent‐Based Discovery of D‐Enantiomeric Antimicrobial Peptides Against Multidrug‐Resistant Bacterial Infection,” Biomaterials 329 (2026): 123927, 10.1016/j.biomaterials.2025.123927. 41443039

[251] H. Ebrahimikondori , D. Sutherland , A. Yanai , et al., “Structure‐Aware Deep Learning Model for Peptide Toxicity Prediction,” Protein Science 33 (2024): e5076, 10.1002/pro.v33.7. 39196703 PMC11193153

[252] B. Maron , J. Rolff , J. Friedman , and Z. Hayouka , “Antimicrobial Peptide Combination Can Hinder Resistance Evolution,” Microbiology Spectrum 10 (2022): e0097322, 10.1128/spectrum.00973-22. 35862981 PMC9430149

[253] B. Ölçeroğlu , A. Filiz , Ş. Çiçek , M. Filiz , A. Katı , and F. B. Barlas , “Nanocarrier‐Enabled Antimicrobial Peptides: Current Progress, Delivery Strategies, and Translational Challenges,” Colloids and Surfaces. B, Biointerfaces 265 (2026): 115679, 10.1016/j.colsurfb.2026.115679. 41946212

[254] S. Zheng , Y. Tu , B. Li , et al., “Antimicrobial Peptide Biological Activity, Delivery Systems and Clinical Translation Status and Challenges,” Journal of Translational Medicine 23 (2025): 292, 10.1186/s12967-025-06321-9. 40055730 PMC11887333

[255] E. Adjei‐Sowah , V. K. Rangasami , A. E. Loiselle , and D. S. W. Benoit , “Optimizing Ligand Valency to Maximize Tendon Accumulation of Peptide‐Targeted Nanoparticles,” ACS Applied Materials & Interfaces 16 (2024): 68864, 10.1021/acsami.4c13388. 39630483 PMC13532700

[256] M. de los A. Ramírez , J. Bou‐Gharios , B. Freis , et al., “Spacer Engineering in Nanoparticle–peptide Conjugates Boosts Targeting Specificity for Tumor‐Associated Antigens,” Nanoscale 17 (2025): 5021–5032, 10.1039/D4NR02931C. 39903198

